# IL-13Rα2 uses TMEM219 in chitinase 3-like-1-induced signalling and effector responses

**DOI:** 10.1038/ncomms12752

**Published:** 2016-09-15

**Authors:** Chang-Min Lee, Chuan Hua He, Adel M. Nour, Yang Zhou, Bing Ma, Jin Wook Park, Kyung Hee Kim, Charles Dela Cruz, Lokesh Sharma, Mahmoud L. Nasr, Yorgo Modis, Chun Geun Lee, Jack A. Elias

**Affiliations:** 1 Department of Molecular Microbiology and Immunology, Brown University, 185 Meeting Street, Box G-L, Providence, Rhode Island 02912, USA; 2 Division of Cardiology, Emory University School of Medicine, Atlanta, Georgia 30322, USA; 3 Section of Pulmonary and Critical Care and Sleep Medicine, Department of Medicine, Yale University School of Medicine, 300 Cedar Street, New Haven, Connecticut 06520, USA; 4 Department of Biological Chemistry and Molecular Pharmacology, Harvard Medical School, 240 Longwood Avenue, Boston, Massachusetts 02115, USA; 5 Department of Medicine, University of Cambridge, MRC Laboratory of Molecular Biology, Francis Crick Avenue, Cambridge CB2 0QH, UK; 6 Division of Medicine and Biological Sciences, Warren Alpert School of Medicine, Brown University, Box G-A1, 97 Waterman Street, Providence, Rhode Island 02912, USA

## Abstract

Recent studies demonstrated that chitinase 3-like-1 (Chi3l1) binds to and signals via IL-13Rα2. However, the mechanism that IL-13Rα2 uses to mediate the effects of Chi3l1 has not been defined. Here, we demonstrate that the membrane protein, TMEM219, is a binding partner of IL-13Rα2 using yeast two-hybrid, co-immunoprecipitation, co-localization and bimolecular fluorescence complementation assays. Furthermore, fluorescence anisotropy nanodisc assays revealed a direct physical interaction between TMEM219 and IL-13Rα2-Chi3l1 complexes. Null mutations or siRNA silencing of TMEM219 or IL-13Rα2 similarly decreased Chi3l1-stimulated epithelial cell HB-EGF production and macrophage MAPK/Erk and PKB/Akt activation. Null mutations of TMEM219 or IL-13Rα2 also phenocopied one another as regards the ability of Chi3l1 to inhibit oxidant-induced apoptosis and lung injury, promote melanoma metastasis and stimulate TGF-β1. TMEM219 also contributed to the decoy function of IL-13Rα2. These studies demonstrate that TMEM219 plays a critical role in Chi3l1-induced IL-13Rα2 mediated signalling and tissue responses.

## Abstract (web summary)

Chitinase 3-like 1 regulates cell death, inflammation and tissue remodelling via IL-13receptorα2. Here, the authors show that TMEM219 is a IL-13Rα2 co-receptor and modulates oxidant-induced apoptosis and lung injury, melanoma metastasis and TGF-β1 signalling, downstream of Chi3l1-IL-13Rα2.

## Graphical abstract

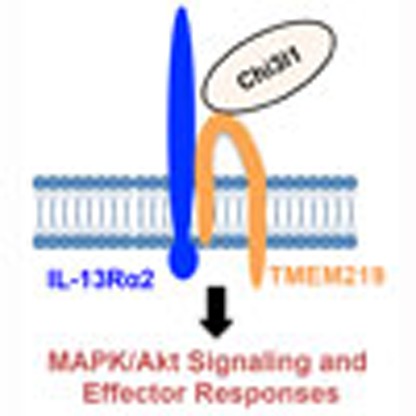

IL-13 Receptor α2 (IL-13Rα2) was originally described as a high affinity receptor for IL-13 that is distinct from the IL-13Rα1-IL-4Rα receptor heterodimer that IL-13 shares with IL-4 (refs 1, 2). It was initially believed to be a decoy receptor for IL-13 because IL-13Rα2 only contains a 17 amino acid cytoplasmic tail that lacks a conserved box 1 region that has been shown to play a critical role in signal transduction^3^, and early studies highlighted its ability to diminish IL-13 responses^1^,^4^,^5^. However, more recent studies have challenged this ‘decoy' concept by demonstrating that IL-13 also signals and regulates a variety of cellular and tissue responses via IL-13Rα2 (refs 2, 6, 7, 8, 9, 10, 11, 12). Recent studies from our laboratory also demonstrated that IL-13 is not the sole ligand for IL-13Rα2 and that chitinase 3-like-1 (Chi3l1, also called YKL-40 in man and BRP-39 in the mouse) binds to, signals and regulates oxidant injury, apoptosis, pyroptosis, inflammasome activation, pathogen responses, melanoma metastasis and TGF-β_1_ elaboration via IL-13Rα2 (ref. 13). These studies also demonstrated that the cytoplasmic tail of IL-13Rα2 was required for the Wnt/β-catenin signalling pathway activation, but not for the activation of MAPK and Akt signaling^13^. However, it is still unclear how IL-13Rα2 accomplishes these varied effector responses. In addition, we are totally lacking in our understanding of how IL-13Rα2 uses its cytoplasmic tail to activate signalling in some settings but not in others. Importantly, the possibility that IL-13Rα2 interacts with a binding partner to mediate many of these effects has not been investigated.

To address the possibility that IL-13Rα2 interacts with other membrane receptors, yeast 2 hybrid (Y2H) screening was employed to identify IL-13Rα2 binding proteins. This was followed by assays to define these interactions and studies using gene silencing, null mutant mice and antibody neutralization to compare the effector roles of IL-13Rα2 and its binding partner. The Y2H screening assay identified transmembrane protein 219 (TMEM219), a known membrane protein, as a molecule that interacts with IL-13Rα2. The studies also demonstrated that these two moieties co-immunoprecipitate and colocalize in double-label immunohistochemistry (IHC), and bimolecular fluorescence complementation (BiFC) assays. Fluorescence anisotropy nanodisc assays also highlighted significant direct interactions between TMEM219 and IL-13Rα2 in the presence of Chi3l1. The gene silencing studies, investigations with newly generated TMEM219 null mice and antibody neutralization evaluations demonstrated that TMEM219 and IL-13Rα2 are both required for Chi3l1-stimulated induction of heparin binding EGF-like growth factor (HB-EGF) by epithelial cells and murine peritoneal macrophages and that TMEM219 interaction with IL-13Rα2 play a critical role in Chi3l1-stimulated activation of MAPK/Erk and Akt/PKB but not Wnt/β-catenin signalling. Lastly, *in vitro* and *in vivo* studies demonstrated that TMEM219 and IL-13Rα2 play similar critical roles in the regulation of cellular apoptosis, the protective effects of Chi3l1 in oxidant-induced cell death and injury responses, lung melanoma metastasis and the induction of total and active TGF-β1. They demonstrated that TMEM219 also contributes to the IL-13 decoy function of IL-13Rα2. When viewed in combination, these studies demonstrate that TMEM219 is an IL-13Rα2 binding partner that plays a critical role in many Chi3l1-induced, IL-13Rα2-mediated cellular and organ responses.

## Results

### TMEM219 binds and co-localizes with IL-13Rα2

**Figure 1:**
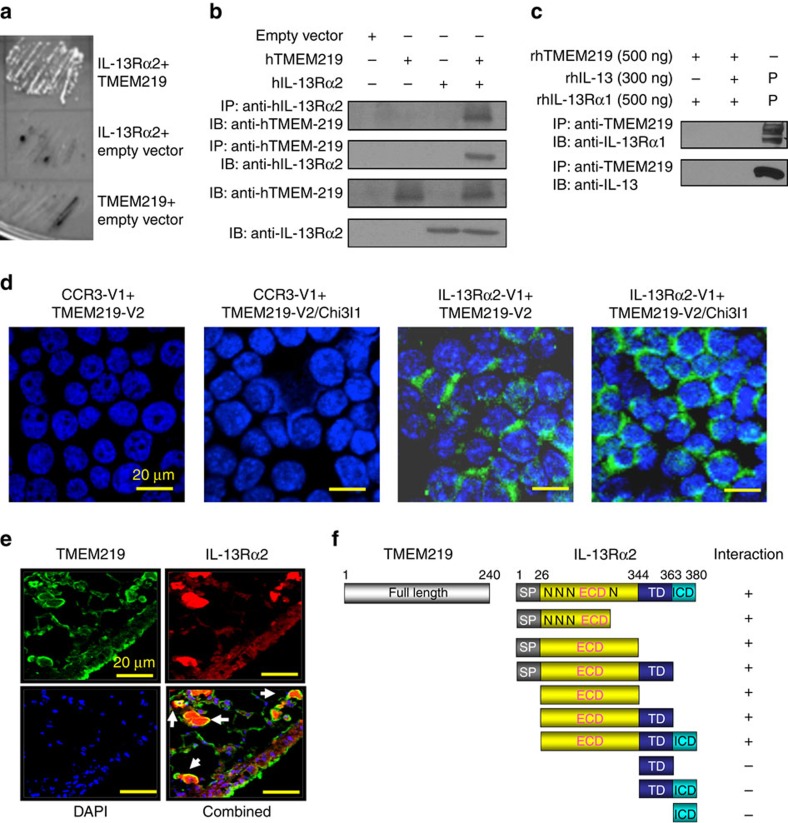
TMEM219 physically interacts with IL-13Rα2. (**a**) Y2H demonstration of the interaction between IL-13Rα2 and TMEM219. (**b**) 1HAEo cells were transfected with hIL-13Rα2 (IL-13Rα2) and/or human TMEM219 (TMEM219) expressing plasmids. Co-IP performed with either anti-IL-13Rα2 (1:500, AF146, R&D) or anti-TMEM219 (1:500, sc-244405, Santa Cruz) antibody, and the precipitates were evaluated using IB analysis as noted. (**c**) Direct interaction between TMEM219 and IL-13Rα1 was tested by Co-IP/IB assay using recombinant human (rh) TMEM219 (350 ng), IL-13 (300 ng, 213-ILB, R&D) and IL-13Rα1 (500 ng, 146-IR, R&D). His-tagged human TMEM219 was expressed and purified in HEK 293T cells using pcDNA 3.1 vector. Commercially available Recombinant human IL-13 and IL-13Rα1 (R&D systems) were used for this assay. (**d**) Interaction of IL-13Rα2 and TMEM219 on cell membrane was confirmed by BiFC assay. IL-13Rα2 and TMEM219 constructs were generated which contain fragments of the tagging protein Venus YFP fragments (V1 and V2). A similar approach was used to generate the negative control, CCR3 containing Venus YFP fragments. The indicated plasmids were transfected into 1HAEo cells, which were treated with rChi3l1 (500 ng ml^−1^, 2599-CH, R&D), rIL-13 (20 ng ml^−1^, 213-ILB, R&D) or vehicle control as noted. (**e**) Immunohistochemical demonstration of the co-localization of IL-13Rα2 and TMEM219 in lungs from IL-13 Tg mice. Fluorescence images were counterstained with 4′6-diamidino-2-phenylindole (DAPI) for nucleus identification. (**f**) Y2H characterization of the IL-13Rα2 sequences critical for binding to hTMEM219. The extracellular domain (ECD), transmembrane domain (TD), intracellular domain (ICD), signal peptide (SP) and sites of N-glycosylation (N) are illustrated. Fragment binding is illustrated with a + sign. (**b**,**c**) Representative of two separate western blot analysis. (**d**,**e**) Composite illustration taken from the noted four groups of mice (*N*>5) that behaved similarly.

To identify the binding partners of IL-13Rα2, Y2H screening was employed as previously described^13^. Multiple candidate molecules were identified when IL-13Rα2 was used as bait against a human lung cDNA library. Surprisingly, 10 of the 19 interacting clones that were obtained encoded TMEM219 (Fig. 1a). The interaction between IL-13Rα2 and TMEM219 was further validated using co-immunoprecipitation (Co-IP) and BiFC assays. In the former, 1HAEo lung epithelial cells were transfected with plasmids containing full-length complementary DNA encoding IL-13Rα2 or TMEM219, subjected to immunoprecipitation with antibodies against one moiety and the precipitate was analyzed using immunoblots (IBs) with antibodies against the other. In these experiments, the two moieties consistently travelled together with IP using antibodies against IL-13Rα2 precipitating TMEM 219 and vice versa (Fig. 1b). Interestingly, TMEM 219 did not interact in a similar manner with IL-13Rα1 (Fig. 1c). The BiFC assays further visualized this interaction in live cells. In these experiments 1HAEo cells were transfected with vectors that contain IL-13Rα2 or TMEM219 each of which contained a partial and complementing yellow fluorescent protein (YFP) construct (Supplementary Fig.1a for detailed map of each construct). When these constructs co-localize, florescence can be appreciated. As shown in Fig. 1d, the fluorescence complementation was apparent after co-transfection of constructs of IL-13Rα2-V1 and TMEM219-V2 while no signals were noted in the cells transfected with individual constructs or cells transfected with individual constructs with negative control constructs (C–C chemokine receptor type 3 (CCR3)-V1 or -V2 or TMEM219-V1 or -V2; Fig. 1d and Supplementary Fig. 1b). Interestingly, when cells were incubated with rChi3l1 (500 ng ml^−1^), a known ligand of IL-13Rα2 (ref. 13), the signal complementation after co-transfection of IL-13Rα2-V1 and TMEM219-V2 was significantly enhanced compared with non-treated cells (Fig. 1d; Supplementary Fig. 1b), suggesting that ligand binding to IL-13Rα2 significantly enhances the physical interaction between IL-13Rα2 and TMEM219. Similar results were seen when primary normal human bronchial epithelial cells (NHBE cells) were transfected with IL-13Rα2-V1 and TMEM219-V2 and treated with rChi3l1 (Supplementary Fig. 1c). In support of the notion that ligands of IL-13Rα2 augment IL-13Rα2-TMEM219 interaction, rIL-13 also increased this interaction in both HAEo transformed cells as well as NHBE cells (Supplementary Fig. 1b,c).

We next localized the expression of TMEM219 and IL-13Rα2 using confocal microscopy and double-label immunostaining. Lungs from IL-13 transgenic (Tg) mice were used in these evaluations because the expression of IL-13Rα2 and TMEM219 are significantly induced in these tissues. We noted that TMEM219 was widely expressed on the outer membranes and to a lesser degree in intracellular locations of the lung cells, with prominent expression in airway epithelial cells and macrophages (Fig. 1e). As we previously demonstrated^13^, IL-13Rα2 was readily appreciated both in an intracellular pool and on the outer membrane of macrophages (Fig. 1e). In all cases this staining was specific because it was not present in the absence of the primary antibody and it was successfully competed against with recombinant peptide. Importantly, TMEM219 and IL-13Rα2 colocalized on the outer cell membranes (Fig. 1e, arrows) but IL-13Rα2 had a more prominent intracellular pool. Lastly, the critical region of IL-13Rα2 that binds to full-length TMEM219 were determined using IL-13Rα2 constructs in the Y2H assay as described previously^13^. These studies demonstrated that the extracellular domain (ECD) of IL-13Rα2 plays a critical role in binding to TMEM219 (Fig. 1f; Supplementary Fig. 1d). When viewed in combination, these studies demonstrate that TMEM219 physically interacts with IL-13Rα2.

### TMEM219 in nanodiscs binds IL-13Rα2-Chi3l1 complexes

**Figure 2:**
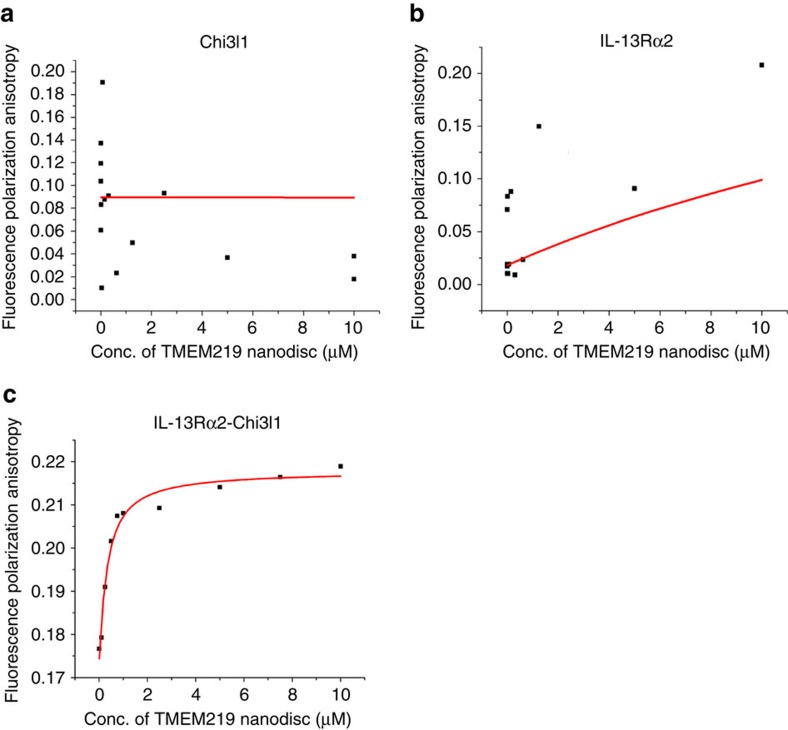
Direct interaction of IL-13Rα2 complexes and TMEM219 nanodisc. (**a**,**b**) Serial dilution of TMEM219 nanodisc (10–10,000 nM) were incubated with 10 nM of BIOPYD-labelled Chi3l1 or IL-13Rα2. No significant increase of fluorescence signals was noted in these nanodisc incubations. (**c**) Serial dilution of TMEM219 nanodisc (10–10,000 nM) were incubated with 10 nM of BIOPYD-labelled IL-13Rα2-Chi3l1 complexes. As the concentration of the nanodisc increases, changes in fluorescence signal increases until it reaches saturation. IL-13Rα2-Chi3l1 complexes directly bind to TMEM219 nanodisc with an affinity of 288 nM, respectively. All the panels are representative of a minimum of two separate experiments.

We further characterized the interactions between TMEM219, IL-13Rα2 and Chi3l1 using nanodisc and fluorescence anisotropy (FA) (also known as fluorescence polarization) assays as described previously^14^. For these evaluations, we expressed Chi3l1 and ectodomains of IL-13Rα2 (ecto-IL-13Rα2) using a baculovirus-insect cell expression system and TMEM219 proteins using a mammalian expression system. Purified TMEM219 protein was reconstituted in 13 nm nanodisc with an estimated stoichiometry of 1 copy per disc (Supplementary Fig. 2a,b). An empty nanodisc was used as a control for nonspecific binding. The changes in FA values obtained from these titrations were close or below the changes in FA of free labelled protein or labelled protein alone (Supplementary Fig. 2c). This demonstrates that the empty nanodisc did not bind to Chi3l1, or IL-13Rα2 complexed with Chi3l1. We next looked for interactions between the ecto-IL-13Rα2, Chi3l1 and the TMEM219 nanodisc. At the concentration range used in this experiment, we did not observe any direct interaction between TMEM219 nanodisc and Chi3l1 or IL-13Rα2 (Fig. 2a,b). However, TMEM219 nanodisc showed strong interactions with IL-13Rα2-Chi3l1 complexes with Kd values of approximately  288 nM (Fig. 2c). These data provided a direct assessment of IL-13Rα2 and Chi3l1 complexing with TMEM219 *in vitro*.

### Roles of TMEM219 and IL-13Rα2 in HB-EGF production

**Figure 3:**
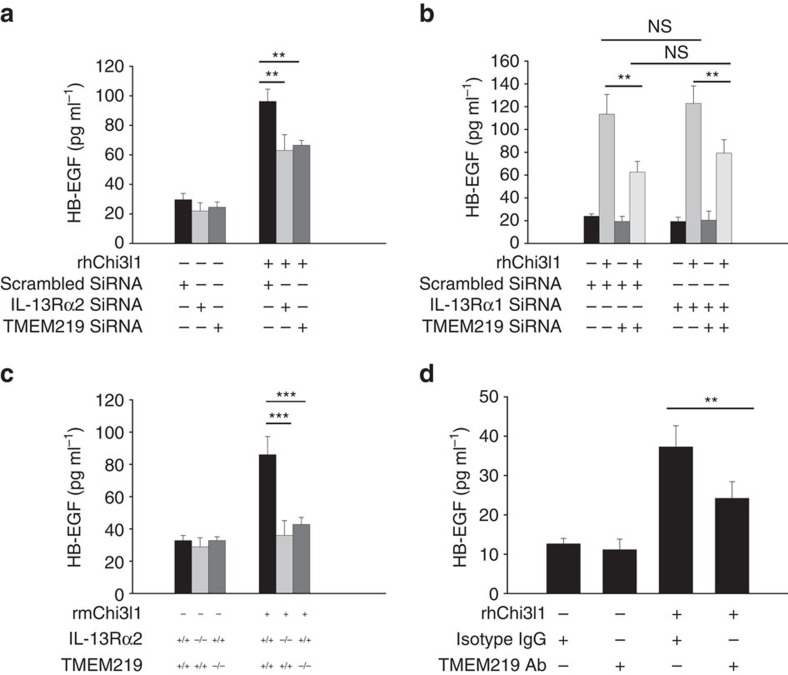
TMEM219 plays a critical role in Chi3l1-stimulated HB-EGF expression by epithelial cells and macrophages. (**a**,**b**) HB-EGF production by 1HAEo cells. 1HAEo cells were treated rhChi3l1 (500 ng ml^−1^) with (+) and without (−) siRNA silencing of IL-13Rα2 or IL-13Rα1 or TMEM219. The concentrations of HB-EGF were assessed by ELISA. (**c**) HB-EGF production by macrophages. Peritoneal macrophages were prepared from WT, IL-13Rα2 or TMEM219 null mutant mice and incubated with rmChi3l1 (500 ng ml^−1^) for 48 h. The levels of supernatant HB-EGF were assessed by ELISA. (**d**) Effects of anti-TMEM219 on HB-EGF production by 1HAEo cells. 1HAEo cells were stimulated for 48 h with rhChi3l1 (500 ng ml^−1^) in the presence and absence of TMEM219 neutralizing antibodies. In all panels, the levels of supernatant HB-EGF were assessed by ELISA. The values represent the mean±s.e.m. of triplicate evaluations in a minimum of three separate experiments. **P<0.01; ***P<0.001; ns, non-significant by Student's *t*-test.

Previous studies demonstrated that human 1HAEo cells produce HB-EGF via an IL-13Rα2-dependent mechanism^11^. In accord with these findings, Chi3l1 also stimulated HB-EGF mRNA accumulation and protein elaboration (Fig. 3a,b; Supplementary Fig. 3a,b). In both cases the silencing of IL-13Rα2 markedly diminished while the silencing of IL-13Rα1 did not alter these inductive events (Fig. 3a,b; Supplementary Fig. 3a,b). Chi3l1 also stimulated HB-EGF mRNA accumulation and protein production by primary mouse macrophages and this induction was also mediated via an IL-13Rα2-dependent mechanism (Fig. 3c; Supplementary Fig. 3c). To determine if TMEM219 played a role in these responses, we evaluated the ability of Chi3l1 to stimulate the expression and production of HB-EGF after siRNA silencing or genetic ablation of TMEM219. The siRNA silencing that was employed was target specific and decreased TMEM219, IL-13Rα2 or IL-13Rα1 expression by approximately >90% (Supplementary Fig. 4). Silencing of TMEM219 in 1HAEo cells and null mutations of TMEM219 in mouse peritoneal macrophages both significantly decreased rChi3l1-stimulated expression of HB-EGF (Fig. 3a–c; Supplementary Fig. 3a–c). Similar decreases in rChi3l1-stimulated HB-EGF expression were noted in the cells treated with monoclonal antibodies against TMEM219 compared with isotype controls (Fig. 3d). Importantly, TMEM219 silencing also caused a similar decrease in Chi3l1-induced HB-EGF production by NHBE cells. When viewed in combination, these studies demonstrate that Chi3l1 stimulates the production of HB-EGF by transformed and normal airway epithelial cells and macrophages via IL-13Rα2-dependent mechanisms and that TMEM219 plays a similarly essential role in these inductive cellular events.

### Roles of TMEM219 and IL-13Rα2 in MAPK and Akt/PKB activation

**Figure 4:**
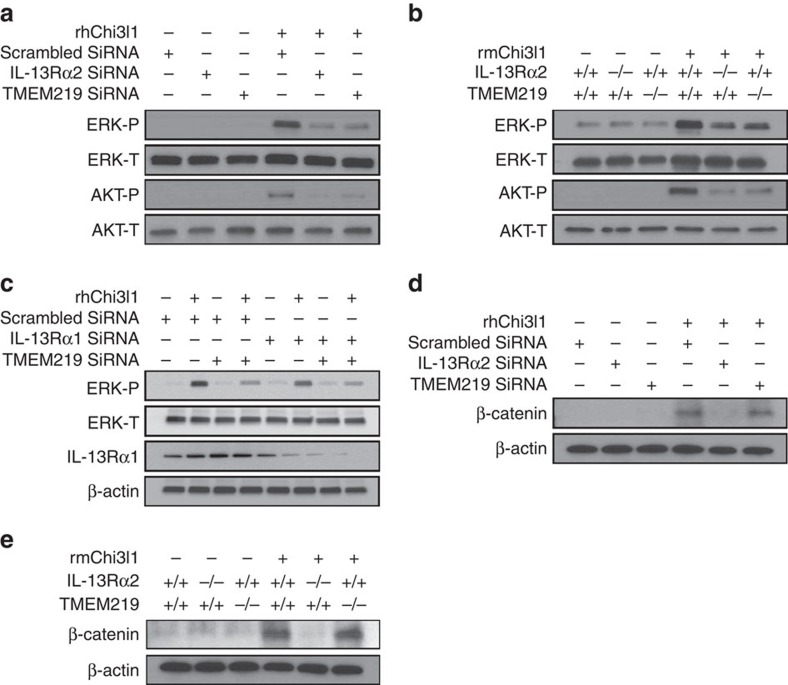
TMEM219 plays a critical and selective role in Chi3l1-stimulated signalling in lung epithelial cells and macrophages. (**a**) 1HAEo cells were stimulated with rhChi3l1 (500 ng ml^−1^) with (+) and without (−) the siRNA silencing of IL-13Rα2 or TMEM219 and western blot evaluations were undertaken to evaluate the activation of the MAPK/ERK and PKB/AKT pathways. (**b**) Peritoneal macrophages from WT, IL-13Rα2 null or TMEM219 null mutant mice were stimulated with rChi3l1 (500 ng ml^−1^, 2649-CH, R&D) for 24 h and western blot evaluations were used to characterize the activation of the MAPK/ERK and PKB/AKT signalling pathways. (**c**,**d**) 1HAEo cells were stimulated with rhChi3l1 (500 ng ml^−1^, R&D) with (+) and without (−) siRNA silencing of IL-13Rα1, IL-13Rα2 or TMEM219 and western blot evaluations of MAPK/ERK activation and β-catenin phosphorylation were undertaken as noted. (**e**) Peritoneal macrophages were isolated from IL-13Rα2 or TMEM219 null mutant mice, stimulated with rmChi3l1 (500 ng ml^−1^, R&D) and western blot evaluations of β-catenin phosphorylation were undertaken. All the panels are representatives of a minimum of three separate experiments.

In previous studies, we demonstrated that Chi3l1 activates MAPK/Erk, Akt/PKB and Wnt/β-catenin signalling via IL-13Rα2-dependent pathways^13^. To evaluate the roles of TMEM219 in these signalling responses, 1HAEo lung epithelial cells and murine peritoneal macrophages were stimulated with rChi3l1 with and without siRNA silencing or null mutations of TMEM219 (Fig. 4a–d). Chi3l1 activated MAPk/Erk and Akt/PKB in 1HAEo lung epithelial cells (Fig. 4a) and murine peritoneal macrophages (Fig. 4b). In both cell types, this activation was IL-13Rα2-dependent because it was significantly decreased by treatment with silencing siRNA or null mutations of IL-13Rα2 (Fig. 4a,b). In contrast, silencing IL-13Rα1 in 1HAEo cells did not alter the ability of Chi3l1 to activate these signalling pathways (Fig. 4c). Similar results were obtained when these cells were made deficient in TMEM219. Specifically, siRNA silencing or null mutations of TMEM219 significantly decreased Chi3l1-stimulated MAPK/Erk and Akt/PKB activation in these cells (Fig. 4a,b). Treatment of 1HAEo cells with neutralizing antibodies against TMEM219 also significantly reduced rChi3l1-stimulated MAPK/Erk and Akt/PKB activation when compared with isotype controls (Supplementary Fig. 5). Importantly, similar decreases in Chi3l1-induced MAPK/Erk and Akt/PKB signalling were seen when TMEM219 was silenced in primary NHBE cells (Supplementary Fig. 6). These studies demonstrate that TMEM219 and IL-13Rα2 play similar and critical roles in Chi3l1- stimulated activation of MAPK/Erk and Akt/PKB.

### Roles of TMEM219 and IL-13Rα2 in Wnt/β-catenin signalling

We previously demonstrated that the intracellular 17 amino acids in IL-13Rα2 play a critical role in Wnt/β-catenin signalling but are not required for MAPK or Akt/PKB activation^13^. In accord with these findings siRNA silencing or null mutations of IL-13Rα2 effectively blocked Chi3l1-induced β-catenin nuclear translocation in 1HAEo cells and murine peritoneal macrophages (Fig. 4d,e). In contrast, ablation of TMEM219 did not affect Chi3l1-stimulated Wnt/β-catenin activation in 1HAEo cells (Fig. 4d) or macrophages (Fig. 4e). These studies demonstrate that, in contrast to the MAPK and Akt signalling pathways, Chi3l1 activates Wnt/β-catenin signalling via a TMEM219-independent mechanism.

### Roles of TMEM219 and IL-13Rα2 in apoptosis and lung injury

**Figure 5:**
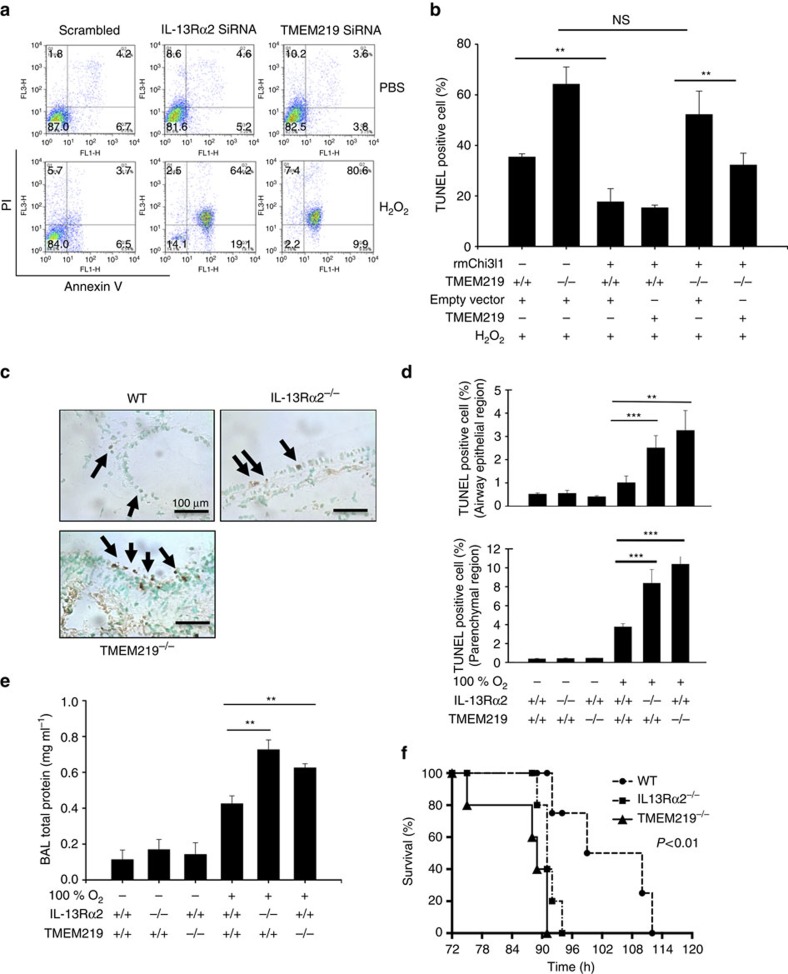
TMEM219 plays a critical role in oxidant-induced cell death *in vitro* and *in vivo*. (**a**) 1 HAEo cells were incubated with hydrogen peroxide (200 μM) or vehicle control for 24 h and cellular apoptosis was evaluated by flow cytometry using Annein-V and PI staining. These experiments were undertaken using 1HAEo cells that had been treated with siRNA that silenced IL-13Rα2 or TMEM219 or appropriate controls (**b**) WT and TMEM219^−/−^ macrophages were incubated in the presence and absence of rhChi3l1. In selected experiments, TMEM219 or an appropriate control was transfected and over expressed (TMEM219+). After 4 h of incubation, the percentage of TUNEL positive cells in three microscopic fields was assessed (× 10). (**c**) WT, IL-13Rα2^−/−^ and TMEM219^−/−^ mice were exposed to 100% oxygen for 72 h, their lungs were collected and cell death was evaluated using TUNEL staining. Representative microscopic images are illustrated. The arrows highlight selected TUNEL positive cells. (**d**) WT, IL-13Rα2^−/−^ and TMEM219^−/−^ mice were exposed to 100% oxygen for 72 h, their lungs were collected and cell death was evaluated using TUNEL staining. The TUNEL positive cells in the airways and parenchymal areas were quantitated by counting at least 10 microscopic fields under × 20 magnification. (**e**) WT, IL-13Rα2^−/−^ and TMEM219^−/−^ mice were exposed to 100% oxygen for 72 h. As an index of lung injury, the levels of total protein in BAL were quantitated. (**f**) Survival analysis of WT (*n*=5), IL-13Rα2^−/−^ (*n*=7) and TMEM219^−/−^ (*n*=7) mice after exposure of 100% O_2_. The TMEM219^−/−^ and IL-13Rα2^−/−^ mice all had significantly decreased survival compared with WT mice (*P*<0.01, log-rank Mantel–Haenszel test). (**a**) Representative of three separate experiments. (**c**) Composite illustration taken from the noted three groups of mice (*N*≥5) that behaved similarly. The values in **b** represent the mean±s.e.m. of triplicate evaluations in a minimum of three separate experiments. The values in **d**,**e** represent the mean±s.e.m. of evaluations in a minimum of five mice **P<0.01; ***P<0.001. NS, not significant by Student's *t*-test.

We recently demonstrated that Chi3l1 inhibits oxidant-induced cellular apoptosis and lung injury via an IL-13Rα2-dependent mechanism(s)^13^. Thus, studies were undertaken to define the role(s) of TMEM219 in these responses. *In vitro* treatment of 1HAEo cells with low dose H_2_O_2_ (200 μM) caused a modest increase in apoptotic cells expressing annexin V and silencing of IL-13Rα2 augmented this cell death response (Fig. 5a). Interestingly, as seen with IL-13Rα2, the silencing of TMEM219 caused a similar significant increase in H_2_O_2_ sensitivity with significantly larger numbers of TMEM219 deficient cells expressing Annexin V compared with cells treated with scrambled siRNA controls (Fig. 5a). Similar results were seen in comparisons of macrophages from wild-type (WT) and TMEM219 null mice. As can be seen in Fig. 5b, the number of cells that were undergoing H_2_O_2_-induced apoptosis, as manifest by positive TUNEL staining, was significantly increased in cells from the TMEM219 null mice versus the controls. In addition, rChi3l1 inhibited H_2_O_2_-induced apoptosis in mice that contained normal levels of TMEM219 (Fig. 5b). In contrast, as previously shown for IL-13Rα2 (ref. 13), the anti-apoptotic effects of Chi3l1 were diminished in the absence of TMEM219 and this defect in Chi3l1-induced antiapoptosis could be rescued by the overexpression of TMEM219 (Fig. 5b). When viewed in combination, these *in vitro* studies demonstrate that, like IL-13Rα2, TMEM219 plays a critical role in the regulation of H_2_O_2_-induced cellular apoptosis.

To define the role of TMEM219 *in vivo*, we characterized the effects of 100% oxygen in WT, IL-13Rα2^−/−^ and TMEM219^−/−^ mice. After 72 h of exposure to hyperoxia, the mice were sacrificed and pulmonary cell death responses were evaluated using TUNEL staining. As previously reported^15^,^16^, hyperoxia caused a tissue cell death response in lungs from WT mice that were characterized by modest numbers of TUNEL positive airway and alveolar epithelial cells, and the magnitude of this response was exaggerated in lungs from IL-13Rα2 null animals (Fig. 5c,d). In accord with the findings in the IL-13Rα2 null mice, a similar increase in airway and parenchymal epithelial cell apoptosis was seen in TMEM219 null animals (Fig. 5c,d). Accordingly, the level of BAL total protein (a measure of oxidant-induced lung injury) was also significantly increased in the IL-13Rα2^−/−^ or TMEM219^−/−^ mice compared with WT controls (Fig. 5e). In addition, with extended exposure to 100% oxygen, the survival of the IL-13Rα2^−/−^ and TMEM219^−/−^ mice was decreased compared with WT controls (Fig. 5f). These studies demonstrate that IL-13Rα2 and TMEM219 play similar roles as inhibitors of oxidant-induced acute lung injury.

### TMEM219 inhibits allergen- and IL-13-stimulated responses

**Figure 6:**
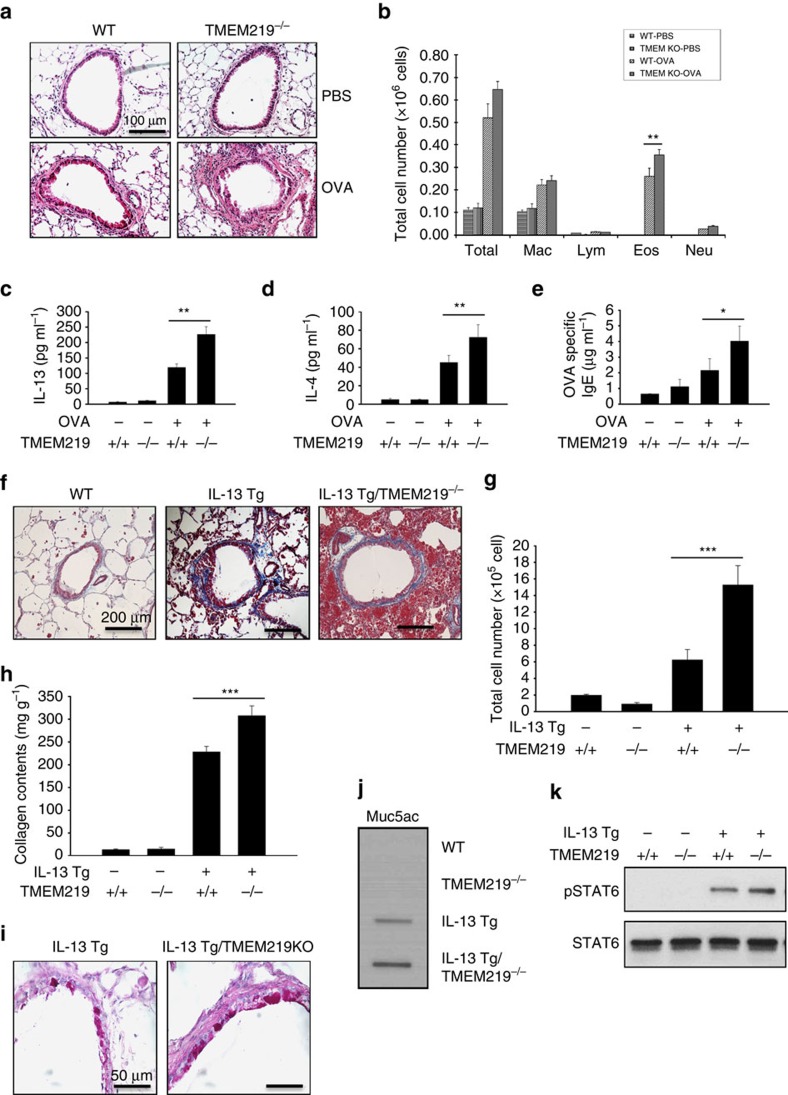
TMEM219 plays critical role in decoy function of IL-13Rα2. (**a**–**e**) Six to eight-weeks-old WT and TMEM219 null (−/−) mice were sensitized and challenged with OVA and the roles of TMEM219 in lung inflammation and cytokine responses were evaluated 24 h after the final allergen challenge. (**a**) Representative lung histology (H&E staining, original magnification × 200). (**b**) BAL cell recovery. (**c**–**e**) Th2 cytokine levels in the BAL measured by ELISA. (**f**–**k**) The roles of TMEM219 in the effector functions of IL-13 were evaluated using the progeny of crosses of IL-13 Tg mice and TMEM219^−/−^ mice. Ten-week-old mice were sacrificed for the histologic evaluations. (**f**) Representative lung sections evaluated with Mallory-trichrome staining. (**g**) Total cell recovery in BAL. (**h**) The levels of lung collagen quantitated by Sircol assay. (**i**) A representative comparison of the levels of D-PAS staining. (**j**) Slot IB evaluation of the levels of BAL Muc5ac. (**k**) Western blot evaluations of the STAT6 activation (phosphorylation) of Tg mice that contain and lack TMEM219. (**a**,**f**,**i**,**j**,**k**) Representative of evaluations in a minimum of five mice per group. The values in (**c**–**e**,**g**,**h**) are the mean±s.e.m. of evaluations on a minimum of five mice. **P<0.01, ***P<0.001. NS, not significant by Student's *t*-test.

IL-13Rα2 was initially described as a decoy receptor that decreased Th2 and IL-13-induced inflammatory and tissue responses^17^. To determine if TMEM219 contributes to the decoy function of IL-13Rα2, we initially evaluated the tissue responses in WT and TMEM219^−/−^ mice after ovalbumin (OVA)-sensitization and challenge. These studies revealed augmented responses in TMEM219 null mice that were similar to what has been described in IL-13Rα2 null animals^18^. Specifically, the tissue infiltration of inflammatory cells, and the levels of BAL eosinophil recovery were significantly increased in TMEM219^−/−^ mice compared with WT controls (Fig. 6a,b). The levels of BAL IL-13, IL-4 and OVA-specific IgE were also significantly higher in TMEM219^−/−^ mice than in similarly treated WT animals (Fig. 6c–e). Interestingly, the levels of IL-1β were not similarly altered (Supplementary Fig. 7a).

Studies were next undertaken to determine if TMEM219 regulated the effects of IL-13. This was done by characterizing the effects of lung-targeted Tg IL-13 in WT and TMEM219 null mice. As shown in Fig. 6, there were impressive increases in IL-13-stimulated fibrotic and inflammatory responses in IL-13 Tg animals that contained null mutations of TMEM219 (IL-13 Tg/TMEM219^−/−^ mice) compared with WT mice and IL-13 Tg mice with WT genetic backgrounds (Fig. 6f–h). In accord with these findings, the levels of total and active TGF-β1 in lungs from IL-13 Tg mice were significantly increased in the absence of TMEM219 (Supplementary Fig. 7b). In addition, IL-13-induced mucus metaplasia and BAL Muc5ac were also increased in the airways of IL-13 Tg/TMEM219^−/−^ mice compared with WT or IL-13Tg only controls (Fig. 6i,j). Lastly, in accord with the known importance of STAT6 signalling in mediating the tissue effects of IL-13 (refs 15, 19), STAT6 activation in lungs from IL-13 Tg mice was also enhanced in mice with null mutations of TMEM219 (Fig. 6k). When viewed in combination, these studies demonstrate that, like IL-13Rα2 (ref. 18), TMEM219 is an important inhibitor of aeroallergen-induced Th2 inflammation and cytokine elaboration and IL-13-induced inflammation, fibrosis, mucus metaplasia and STAT6 signalling.

### TMEM219 contributes to the decoy effects of IL-13Rα2

The studies noted above demonstrate that TMEM219 inhibits IL-13-induced tissue responses in a manner that is similar to the decoy function attributed to IL-13Rα2. To address this possibility more formally, we first assessed the *in vivo* association of IL-13 and IL-13Rα2 in the presence and absence of TMEM219. In these studies we used Co-IP and IB assays with lung lysates from progeny of crosses of IL-13 Tg mice and TMEM219^−/−^ mice. These studies highlighted decreased levels of binding between IL-13 and IL-13Rα2 in the lungs from IL-13Tg mice that lacked TMEM219 compared with IL-13Tg with a WT background (IL-13Tg+/TMEM^+/+^) (Supplementary Fig. 8a). We also evaluated the ability of rIL-13 to bind to rIL-13Rα2 in the presence and absence of rTMEM219. These studies demonstrated that ability of rIL-13 to bind to rIL-13Rα2 was increased in the presence of rTMEM219 while rTMEM219 did not bind to rIL-13 (Supplementary Fig. 8b,c). These findings demonstrate that TMEM219 facilitates IL-13-IL-13Rα2 binding and by so doing contributes to the decoy function attributed to IL-13Rα2.

### TMEM219 in melanoma metastasis and TGF-1 production *in vivo*

**Figure 7:**
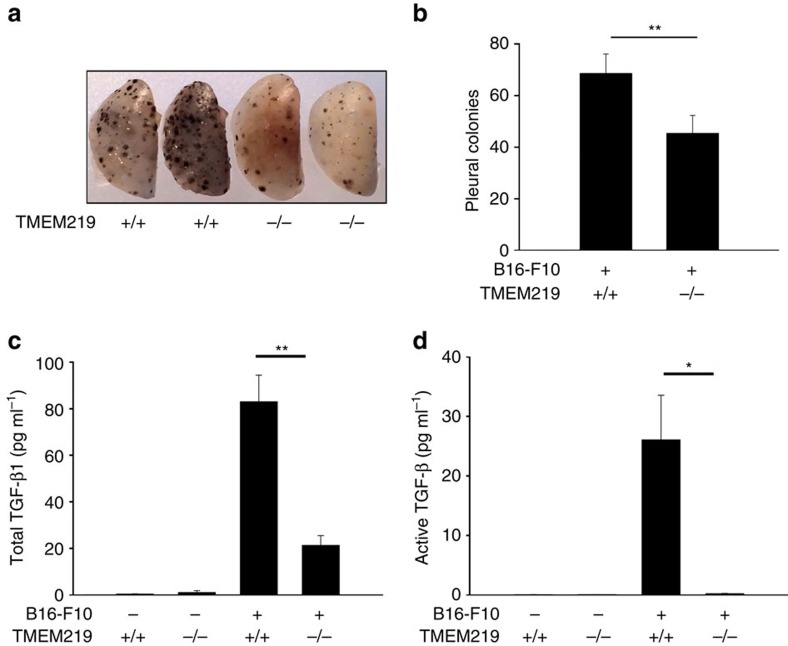
TMEM219 plays a critical role in lung melanoma metastasis. TMEM219 null (−/−) mice and WT (+/+) mice were challenged with B16-F10 melanoma cells and lung metastasis was evaluated. (**a**) Representative photograph of lungs from WT and TMEM219 null mice 2 weeks after challenge with B16-F10 melanoma cells. (**b**) The number of pleural melanoma colonies in lungs from melanoma-challenged WT and TMEM219 null mice. (**c**,**d**) The levels of total and active TGF-β1 in BAL fluids from WT mice and TMEM219^−/−^ mice. (**a**) Representative photograph of lungs from an experiment with a minimum of five mice per group. The values in the remaining panels represent the means±s.e.m. of evaluations in a minimum of four mice. *P<0.05; **P<0.01 by Student's *t*-test.

Recent studies from our laboratory and others have demonstrated that the metastasis of malignant melanocytes to the lung is mediated by an IL-13Rα2-dependent mechanism, which requires the production of TGF-β1 (refs 2, 10, 13). Thus, studies were undertaken to determine if TMEM219 plays a role in these responses. To accomplish this, we compared the metastasis of B16-F10 melanoma cells and the TGF-β1 that they induce in WT mice and TMEM219 null mice. Melanoma cell administration caused impressive levels of metastasis in lungs from WT mice and this metastatic response was markedly decreased in lungs from TMEM219^−/−^ mice (Fig. 7a,b). In accord with the studies of Strober *et al.*^2^,^10^ melanoma metastasis was associated with significant increases in the levels of total and activated TGF-β1 in lungs from WT mice (Fig. 7c,d). Importantly, the levels of TGF-β1 were significantly decreased in mice with null mutations of TMEM219 (Fig. 7c,d). When viewed in combination, these studies demonstrate that, like IL-13Rα2, endogenous TMEM219 plays a critical role in pulmonary melanoma metastasis and the TGF-β1 elaboration that underlies this response.

## Discussion

IL-13Rα2 is a 56 kDa glycoprotein that has engendered controversy based on its reported ability to act as a decoy as well as a signalling receptor^3^,^8^,^13^,^17^. To further understand the biology of IL-13Rα2 and shed light on this controversy, studies were undertaken to determine if this complex biology might be able to be explained, at least in part, by the ability of IL-13Rα2 to interact with and utilize binding partners in mediating ligand-induced effector responses. These studies demonstrate that IL-13Rα2 binds to TMEM219 at baseline and that this interaction is augmented in the presence of the IL-13Rα2 ligands Chi3l1 and IL-13. They also demonstrate that optimal Chi3l1 activation of MAPK and Akt signalling, inhibition of apoptosis, amelioration of oxidant injury, mediation of melanoma metastasis, stimulation of TGF-β1 elaboration during lung metastasis and inhibition of Th2 and IL-13-induced inflammation require both IL-13Rα2 and TMEM219.

To begin to address the possibility that IL-13Rα2 interacts with other proteins in mediating its effector responses, we initially employed Y2H screening to identify molecules that bind to IL-13Rα2. These studies identified TMEM219 as an IL-13Rα2 binding partner. This was followed by studies that demonstrated that IL-13Rα2 and TMEM219 co-immunoprecipitate and colocalize. Furthermore, reconstitution of purified TMEM219 into 13 nm nanodiscs and FA experiments confirmed the interaction of the TMEM219 nanodiscs and the purified ecto-IL-13Rα2 in the presence of Chi3l1. These observations allow for the interesting possibility that IL-13Rα2 may first bind its ligand, Chi3l1 or IL-13, and that this complex is then able to recruit TMEM219 to initiate signalling. Interestingly, both IL-13Rα2 and TMEM219 have short intracellular tails. This allows for the interesting hypothesis that IL-13Rα2 and TMEM219 behave like the α and β dimers of the T cell antigen receptor (TCR), which also have short intracytoplasmic tails and utilize other surface molecules like CD3 to transmit signals^20^,^21^. IL-13Rα2 and TMEM 219 both exist in the cell membrane and in intracellular pools. Thus, additional investigation will be required to further define the mechanisms that IL-13Rα2 and TMEM219 use to transmit signals and the locations and constituents of this IL-13Rα2 and TMEM219 containing receptor complex.

Previous studies from our laboratory demonstrated that Chi3l1 binds to and signals via IL-13Rα2 (ref. 13). In these studies, it was appreciated that Chi3l1 interacts with IL-13Rα2 via different mechanisms to activate different downstream signalling pathways. Specifically, the Wnt/β-catenin pathway and the MAPK and AKT pathways were activated via mechanisms that were independent of and dependent on the 17 amino acid intracellular segment of IL-13Rα2, respectively^13^. The present studies confirm these observations by once again showing differences in the ways that IL-13Rα2 activates these signalling pathways. They also provide a potential mechanism for these differences by demonstrating that IL-13Rα2 activation of MAPK/Erk and Akt are dependent on TMEM219 while the Wnt/β-catenin pathway activation is mediated via an alternative mechanism. It will be interesting to determine if IL-13Rα2 is able to activate the Wnt/β-catenin pathway by itself or if it uses other binding partners to accomplish this event.

In keeping with its evolutionary conservation over species and time, Chi3l1 has been shown to regulate a number of fundamental processes including the cell death responses, tissue oxidant injury, tumour metastasis and the induction of TGF-β1 and HB-EGF elaboration^13^,^16^,^22^,^23^,^24^. In accord with prior reports from our laboratory, these responses were mediated via pathways that required IL-13Rα2 (ref. 13). Importantly, each also required TMEM219 because phenocopies were seen in mice that lacked IL-13Rα2 or TMEM219 and cells in which each of these moieties was silenced. When viewed in combination, these studies demonstrate that signalling via the IL-13Rα2 and TMEM219 containing multimeric chitosome is an important event in these critical and diverse biologic responses. They also suggest that interventions that target IL-13Rα2, TMEM219 or other components of the chitosome might be useful in attempts to control these fundamental biologic events.

IL-13Rα2 was initially described as a decoy receptor that controls the tissue effects of IL-13. Subsequent studies demonstrated that IL-13Rα2 is a signalling receptor that mediates important biologic events^2^,^8^. In seeming support of the latter point of view, the present studies demonstrate, for the first time, that IL-13Rα2 utilizes TMEM219 as a partner in many of these responses. To address the possibility that TMEM219 also plays a role in the decoy function of IL-13Rα2, we compared the adaptive Th2- and IL-13-induced responses in WT mice and mice with null mutations of TMEM219. In accord with observations in IL-13Rα2 null mice^18^, we noted augmented aeroallergen-induced and IL-13-induced inflammation, cytokine, IgE, remodelling and signalling responses in mice that lacked TMEM219. Studies with pulmonary tissues and recombinant proteins also showed that TMEM 219 contributes to optimal IL-13 binding to IL-13Rα2. These studies demonstrate that TMEM219 plays an important role in the decoy as well as the signalling properties of IL-13Rα2. They also contribute to the resolution of the controversy regarding the fundamental role of IL-13Rα2 by demonstrating that the decoy and signalling features of IL-13Rα2 are not mutually exclusive and that both are important features of this moiety.

The present studies demonstrate that mice that lack IL-13Rα2 or TMEM219 manifest similar increases in IgE production and Th2 cytokine elaboration and similar decreases in MAPK and Akt signalling and melanoma metastasis. They also demonstrate that TMEM219 and IL-13Rα2 bind to each other, that TMEM219 is required for optimal Chi3l1-induced, IL-13Rα2-dependent cell and tissue effects and that TMEM219 augments the binding of IL-13 to IL-13Rα2 without interacting directly with IL-13Rα1. As noted above, these findings demonstrate that TMEM 219 plays a critical role in Chi3l1 signalling while simultaneously contributing to the decoy effects of IL-13Rα2. They also suggest that, in the absence of TMEM219, the local levels of free IL-13 are increased thereby augmenting IL-13 signalling via the canonical IL-13 receptor dimer (IL-13Rα1 and IL-4Rα). This explains how an absence of TMEM219 induces tissue responses characterized by decreased MAPK/Erk and Akt signalling and increased Stat6 signalling. It also suggests that interventions that block the interaction of IL-13Rα2 and TMEM219 will be powerful regulators of inflammation, injury and or remodelling. It will be exciting to test these interventions in diseases and models of diseases that are characterized by Chi3l1 excess once they are developed.

The biology of TMEM219 has not been a topic of extensive investigation. The little that has been done has identified TMEM219 as a receptor for insulin-like growth factor binding protein-3 (IGFBP-3)^25^ and demonstrated that it contributes to biologic responses that are IGFBP3-dependent and -independent^25^,^26^. In these settings, TMEM219 has been shown to inhibit cell proliferation and induce cellular apoptosis, activate MAP kinases and induce angiogenesis^25^,^27^,^28^,^29^,^30^. Interestingly, the proapoptotic effects of TMEM219 that have been described to date stand in marked contrast to the IL-13Rα2 and TMEM219-mediated anti-apoptotic effects of Chi3l1 that are described in this report. In contrast, there are interesting similarities between the TMEM219-based activation of MAP kinases described in the literature and in this report. In addition, proangiogenic properties of Chi3l1 have also been described^31^. When viewed in combination, it is tempting to speculate that TMEM219 has ligand and cell context specific effector functions and that the presence of IL-13Rα2, and its ligands is a major determinant of the role of TMEM219 in a given setting. It is also tempting to speculate that the angiogenic effects of TMEM219 that have been described to date are mediated via its ability to interact with IL-13Rα2 and its ligands. Additional investigation will be required to address these speculations.

Prior studies from our laboratory and the present investigations demonstrate that IL-13Rα2 and TMEM219 play critical roles in melanoma metastasis^13^. In accord with the studies of Strober *et al.*^2^,^10^ that demonstrated that melanoma metastasis requires the induction of TGF-β1 in lungs from WT mice, the levels of TGF-β1 were significantly decreased in mice with null mutations of TMEM219 (Fig. 7c,d). In contrast, studies from our laboratory and others demonstrated that Tg IL-13 elicits tissue fibrosis via mechanisms that are dependent and independent of TGF-β1 (refs 32, 33, 34) and the present studies demonstrate that, in the absence of TMEM219, the levels of TGF-β1 and tissue fibrosis are increased. These findings are in accord with our prior demonstration that the levels of TGF-β1 are increased in IL-13 Tg mice with null mutations of IL-13Rα2 when compared with identical Tg mice on a WT background^18^. They are also in accord with our demonstration that TGF-β1 induction in IL-13 Tg mice is mediated by the canonical IL-13 receptor (IL-13Rα1-IL-4 Rα) while TGF-β1 induction in the setting of melanoma metastasis is mediated via an IL-13Rα2-dependent mechanism. When viewed in combination, these studies allow for the interesting speculation that the increased fibrosis that is seen in IL-13 Tg mice with null mutations of TMEM219 is due to the abrogation of IL-13Rα2 decoy function with heightened levels of free IL-13 and exaggerated IL-13-induced tissue effects including TGF-β1 elaboration. These studies also highlight the complex relationships between IL-13, the IL-13 receptors and TGF-β1 and the context-dependent way that TMEM219 contributes to these responses since it plays a critical role in TGF-β1 elaboration in the presence of metastasis but inhibits TGF-β1 in the presence of Tg (free) IL-13.

In summary, these studies demonstrate that TMEM219 binds IL-13Rα2 complexed with Chi3l1 and that the multimeric complex that is formed mediates diverse signalling and effector responses of Chi3l1. They also highlight the importance of TMEM219 in key Chi3l1-induced and IL-13Rα2-mediated biologic responses including the regulation of cellular apoptosis, oxidative tissue injury, allergic inflammation, tumour metastasis and TGF-β1 and HB-EGF elaboration. Additional investigation of TMEM219, IL-13Rα2, Chi3l1 and the multimeric chitosome complex that they form is warranted in health and disease.

## Methods

### Mice

C57BL/6 mice were purchased from the Jackson Laboratory (Bar Harbor, ME, USA) and were housed at the Yale University and Brown University animal facilities. IL-13 Tg mice were maintained and characterized in our laboratory as previously described^22^. IL-13Rα2 null mice (IL-13Rα2^−/−^) were purchased from The Jackson Laboratory and backcrossed to C57BL/6 background (>10 generations). TMEM219 null mice (TMEM219^tm1(KOMP)Vlcg^) were purchased from The Knock Out Mice Project (KOMP) Repository at University of California, Davis. The mice that were generated were backcrossed to a C57BL/6J background for >10 generations and age- and sex-matched 6–8-weeks-old mice were used for proposed experiment. All murine procedures were approved by the Institutional Animal Care and Use Committees at Yale and Brown Universities.

### Y2H screening

The full-length human IL-13Rα2 gene was amplified by PCR from human lung cDNA using the following primers (forward, 5′-AAG GTT TTCCAT ATG (Nde1) GGA GAA ATG GCT TTC GTT TGC-3′; reverse, 5′-GTC TCT TGA TAT CTC GAG (Xho1) TCT TCA TGT ATC ACG GAA-3′). The IL-13Rα2 DNA was cloned into the Y2H BD vector at the Nde1 and Sal1 sites. The Matchmaker System 3 two-hybrid assay using *S. cerevisiae* (Clontech, Palo Alto, CA, USA) was used to detect interactions between IL-13Rα2 and other cellular proteins. *S. cerevisiae* strain AH109 (Clontech) containing the four reporter genes *ADE2*, *HIS3*, *MEL1* and *lacZ* was co-transfected with pGBKT7-IL-13Rα2 and human lung cDNA library (Clontech) constructed into the vector pAC2 by the lithium acetate method. AH109 cells were grown on plates containing medium lacking tryptophan and leucine (Trp^–^ Leu^–^) to select positive clone(s) containing both IL-13Rα2 (as a bait) and library plasmids. A total of 2 × 10^6^ of these double transformants were then grown on dropout medium deprived of tryptophan, leucine, histidine and adenine (Trp^–^ Leu^–^ His^–^ Ade^–^) to select for protein–protein interactions. Transformants that grew were assayed for α-galactosidase activity on 5-bromo-4-chloro-3-indolylphosphate (X-αβGal) plates according to the manufacturer's instructions (Clontech, Palo Alto, CA, USA). Plasmids were extracted from blue yeast clones and were then transfected into *Escherichia coli* DH5α. Bacteria were grown on medium with ampicillin to select for bacteria containing library plasmids. The plasmid DNA was then isolated and sequenced with a primer of the pGAD-T7 cloning vector and a primer reverse complementary of the pGAD-T7 vector as a forward and reverse primer, respectively. Homology searches were performed using the National Center for Biotechnology Information database with Mega BLAST. All positive constructs were rescreened for their ability to grow on complete dropout medium when transfected together with IL-13Rα2 bait or with the empty pGBKT7 plasmid to verify potential interactions and to eliminate false positives.

### Cell culture

1HAEo human airway epithelial cells were obtained from Dr. Dieter Gruenert, (UCSF, San Francisco, CA, USA). Normal human bronchial epithelial cells (NHBE cells) were obtained from the American Type culture Collection (ATCC; Rockville MD, USA). The authentification of 1HAEo cells and mycoplasma infection were tested with the commercial services provided by ATCC (ATCC # 135-XV and 119-X for STR profiling service and mycoplasma testing, respectively).

### Co-IP and IB analysis

To assess interactions between IL-13Rα2 and TMEM219, epithelial cells were co-transfected with IL-13Rα2 and TMEM219 plasmids (pcDNA3.1). Lysates from these cells were subjected to immunoprecipitation using anti-hIL-13Rα2 mouse monoclonal antibody (1/500 dilution, AF146, R&D system, Minneapolis, USA) or anti-TMEM219 polyclonal antibody (1/500 dilution, sc-244404, Santa Cruz Biotechnology Inc., CA, USA). The Catch and Release V2.0 (Reversible Immunoprecipitation System, EMD Millipore) kit was used for Co-IP according as per the manufacturer's instruction. The precipitates were then evaluated by immunoblotting with antibodies against IL-13Rα2 or TMEM219, respectively. Full scan of all the IBs are available in Supplementary Fig. 9.

### BiFC assay

The full-length human IL-13Rα2 cDNA was generated by PCR amplification from a lung cDNA library using following primers (forward, 5′-TAT ATA TAT GGC GGC CGC (Not1) ATG GCT TTC GTT TGC TTG GCT ATC GGA TGC TTA TAT-3′; reverse, 5′-GCG GCG GCG ATC GAT (ClaI) TGT ATC ACA GAA AAA TTC TGG AAT CAT TTT TGG GTA GGT-3′). The IL-13Rα2 DNA was cloned into the pcDNA3.1/Zeo(+) plasmid containing YFP(V1) (V1 vector) at the Not1 and Cla1 sites. The full-length human TMEM219 cDNA was amplified by PCR from a lung cDNA using the following primers (forward, 5′-TAT TAT TAT AGC GGC CGC (Not1) ATG GGC AAC TGC CAG GCA GGG CAC AAC CTG CAC CTG-3′; reverse, 5′-TAT TAT ATG ATC GAT (ClaI) GAG CCG GGT TCT AGA CCA GTG GGA CTC CCG GCG CGG GTG-3′). TMEM219 was also cloned into the pcDNA3.1/Zeo(+) plasmid containing YFP(V2) (V2 vector) at Not1 and Cla1 sites. Two non-fluorescent fragments of IL-13Rα2-V1 and TMEM219-V2 can form a green fluorescent complex when they are fused each other. For these evaluations, epithelial cells co-transfected with these two cloned plasmids were treated with recombinant IL-13 (20 ng ml^−1^) for 2 h and subjected to fluorescence microscopy 24–72 h after transfection using a Leica TCS SP2 confocal microscope. Fluorescence-activated cell sorting (FACS) evaluation was performed to quantify the cells with green fluorescence using FACScalibur (Becton Dickinson) and FACScalibur software (Becton Dickinson, San Jose, CA, USA).

### Nanodisc and FA assay

To determine if the membrane proteins TMEM219 and IL-13Rα2 directly interact with one another, we employed the FA assay as described previously^14^. IL-13Rα2, Chi3l1 and IL-13 were expressed in insect cells and purified according to the methods previously described in our laboratory^13^. His-tagged TMEM219 was cloned into pcDNA3.1 vector and expressed in HEK293T cells. TMEM219 was purified using Nickel beads (Qiagen). A nanodisc is composed of nanometer-sized phospholipid bilayer encircled by two copies of α-helical amphipathic membrane scaffold proteins (MSPs)^35^. Reconstituted membrane protein into a nanodisc makes the protein water-soluble and provides an effective approach to study it^35^. This method overcame the problems reported with binding assays in living cell, purified membrane protein in detergent, and the heterogeneity problem of reconstituting membrane protein into liposomes^36^,^37^,^38^,^39^. Protein labelling in FA assay was done using BODPY-TRM-X-succinimidyl ester (Invitrogen) in phosphate-buffered saline (PBS) according to the provider's recommendations. Serial dilutions of TMEM219 nanodisc (10 nM–10 μM) were used to measure the binding of BODPY-TRM-X-labelled ectodomain of IL-13Rα2 alone or IL-13Rα2 complexed with IL-13 or Chi3l1. A control for nonspecific binding, we used the empty nanodisc, a nanodisc with the same lipid composition of TMEM219 nondisc with MSPs. FA measurement was done using a QuantaMaster cuvette-based spectrofluorometer (Photon Technology International, NJ, USA). Each sample was run in triplicates and an average of 60 readings was used for calculation. Binding constants (Kds) were determined with Origin 7 (OriginLab, MA, USA) by fitting the data as described 40.

### Co-localization with double-label IHC

To localize the expression of IL-13Rα2 and TMEM219, double-label IHC was undertaken with a modification of procedures described previously by our laboratory^22^. First slides were deparaffinized in xylene, rehydrated in an ethanol gradient, and washed in PBS. To unmask antigens, slides were placed for 20 min at high temperature under high pressure in citrate buffer (10 mM sodium citrate, 0.05% Tween 20, pH 6). Tissue sections were then blocked with a non-serum protein-blocking reagent (DakoCytomation Inc., Mississauga, ON, Canada) for 15 min at room temperature and incubated with primary antibodies (anti-IL-13Rα2 (1/50 dilution, AF539, R&D systems), anti-TMEM219 (1/50 dilution, sc-244404, Santa Cruz Biotechnology Inc.) for 60 min at room temperature in a humid chamber. Substitution of the primary antibody with PBS served as a negative control. The slides were then washed in PBST (0.01 % Tween 20), incubated with secondary antibodies conjugated with horseradish peroxidase (Santa Cruz Biotechnology Inc., Santa Cruz, CA, USA). Reaction products were developed using diaminobenzidine (DAB) containing 0.3% hydrogen peroxide up to 5 min. Cell nuclei were stained with Mounting Medium with DAPI (50 μl, H-1200, VECTOR laboratories, CA, USA). Fluorescence was detected by Immunofluorescence microscopy.

### Preparation and stimulation of peritoneal macrophages

Peritoneal macrophages were isolated from 7-week-old IL-13Rα2 null, TMEM219 null and WT mice, which had been injected intraperitoneally 3 days earlier with 3 ml of thioglycollate. The collected cells were washed twice with PBS and then cultured in DMEM containing 10% heat-inactivated FBS, 1 mM glutamine, 100 of IU ml^−1^ penicillin and 0.1 mg ml^−1^ of streptomycin at a density 2 × 10^6^ cells per ml. The cells were then allowed to adhere for 3 h to a 24-well culture flask at 37 °C in a 5% CO_2_ incubator. Then, the cultures were washed twice with PBS to remove non-adherent cells before the addition of 1 ml of fresh medium. Antibodies against F4/80 (565410, PharMingen, San Diego, CA, USA) and FACS analysis on a FACSCaliber apparatus (Becton Dickinson, San Jose, CA, USA) were used to assess cellular purity. In select experiments macrophages were incubated with IL-13, rChi3l1 or vehicle control. Experiments with rChi3l1 were done in the absence of serum.

### siRNA silencing of IL-13Rα2 and TMEM219

siRNAs specific for human IL-13Rα2 and TMEM219 (Santa Cruz) were used to decrease the levels of mRNA encoding IL-13Rα2 and TMEM219 as per the manufacturer's instructions. Cells were seeded on six-well plates and transfected the next day with IL-13Rα2, TMEM219 or control siRNAs. The cells were collected at the indicated time points and were subjected to real-time (RT)-PCR or western blot evaluations. Quantitative RT-PCR and western blot evaluations using anti-IL-13Rα2 (1/500 dilution, AF146, R&D systems) and anti-TMEM219 (1/500 dilution, sc-244404, Santa Cruz Biotechnology Inc.) antibodies demonstrated that these siRNA had over 90% of silencing efficacies for IL-13Rα2 and TMEM219 (Supplementary Fig. 4).

### Generation of monoclonal antibodies against TMEM219

Anti-mouse TMEM219 monoclonal antibody was generated using synthetic 10-mer peptides (RPEFHWSRTR corresponding to 230–239 region of TMEM219) (Abmart, Shanghai, China) and further purified using Montage Antibody Purification Kit with PROSEP-G Media (EMD Millipore Corporation, Billerica, MA, USA).

### Administration of melanoma cells

The mouse melanoma cell line (B16-F10) established from C57BL6/J mouse melanoma was purchased from ATCC (Cat#: CRL-6475). After being cultured to confluence in Dulbecco's Modified Eagles Medium (DMEM), the cells were collected, adjusted to a concentration of 10^6^ cells per ml and delivered to the mice by tail vein injection (2 × 10^5^ cells per mouse in 200 μl of DMEM). Two weeks after melanoma cell challenge, lungs from these mice were collected and melanoma colonies were counted according to the procedures described in our laboratory^24^.

### Oxidant-induced cell death response *in vitro* and *in vivo*

The cell death and DNA injury that were induced by *in vitro* exposure of 1HAEo lung epithelial cells to H_2_O_2_ (200 μg ml^−1^, J.T. Baker Chemical, NJ, USA) were evaluated with TUNEL staining and FACS analyses of Annexin V and propidium iodide (PI) staining (Kit Cat # 88-8005, ebioscience) as described by our laboratory^22^. These evaluations were undertaken in the presence and absence of rmChi3L1 (R&D Systems, 500 ng ml^−1^), after treatment with TMEM219 and or control siRNA (Santa Cruz Biotechnology Inc., Santa Cruz, CA, USA) or after transfection with TMEM219 or control cDNA. The *in vivo* cell death response was evaluated in WT, TMEM219^−/−^ and IL-13Rα2^−/−^ mice after exposure to 100% oxygen or room air for up to 3 days as described previously^16^.

### Data availability

All data supporting the findings of this study are available within the article and its Supplementary Information files or are available from the corresponding author upon request.

## Additional information

**How to cite this article:** Lee, C.-M. *et al.* IL-13Rα2 uses TMEM219 in chitinase 3-like-1-induced signalling and effector responses. *Nat. Commun.* 7:12752 doi: 10.1038/ncomms12752 (2016).

## Supplementary Material

Supplementary Information Supplementary Figures 1-9
