# Corrigendum: IL-13Rα2 uses TMEM219 in chitinase 3-like-1-induced signalling and effector responses

**DOI:** 10.1038/ncomms13541

**Published:** 2016-11-14

**Authors:** Chang-Min Lee, Chuan Hua He, Adel M. Nour, Yang Zhou, Bing Ma, Jin Wook Park, Kyung Hee Kim, Charles Dela Cruz, Lokesh Sharma, Mahmoud L. Nasr, Yorgo Modis, Chun Geun Lee, Jack A. Elias

*Nature Communications* 7: Article number: 12752 ; DOI: 10.1038/ncomms12752 (2016); Published 09 15 2016; Updated 11 14 2016

The financial support for this Article was not fully acknowledged. The acknowledgements should have included the following:

Yorgo Modis, PhD was supported by a senior research fellowship from the Wellcome Trust, grant no. 101908/Z/13/Z.

