# Supplementary material for: IL-13Rα2 uses TMEM219 in chitinase 3-like-1-induced signalling and effector responses
Source: Nat Commun. 2016 Sep 15;7:12752. doi: 10.1038/ncomms12752 (PMC5027616; doi:10.1038/ncomms12752)
Supplement: Supplementary Information — Supplementary Figures 1-9 [file ncomms12752-s1.pdf]

Supplementary Figure 1

a

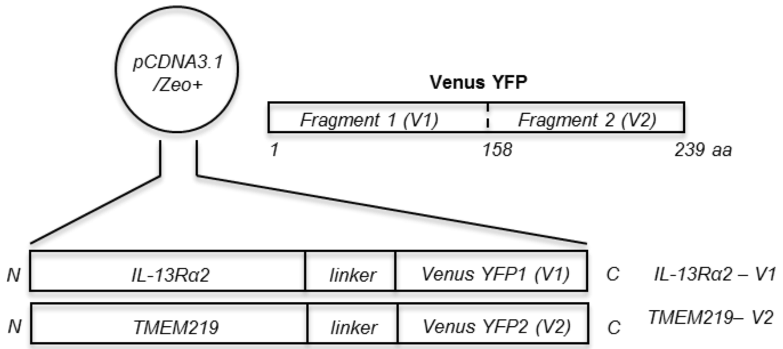

b

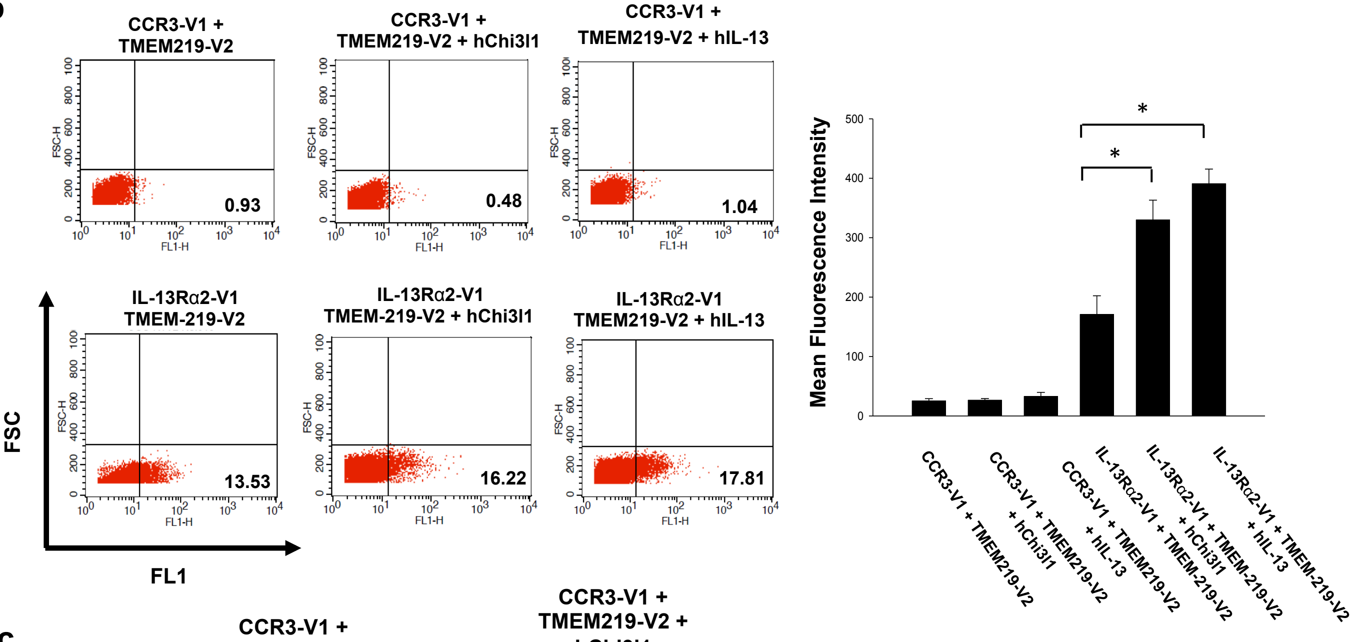

c

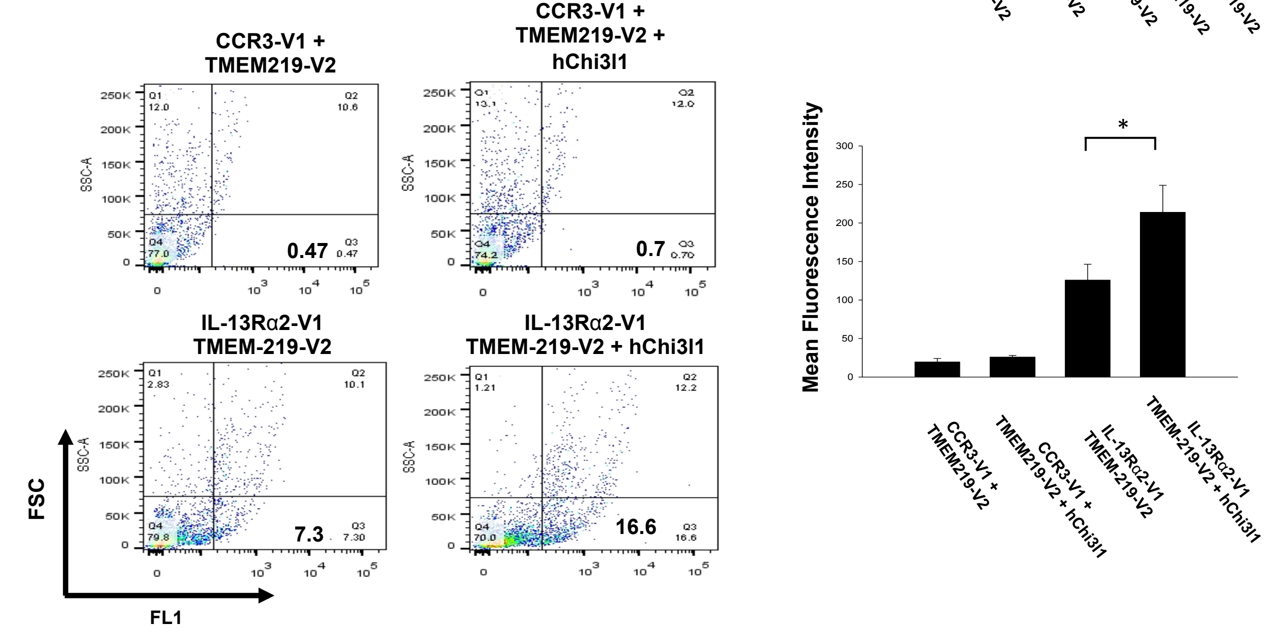

Supplementary Figure 1 continued

d

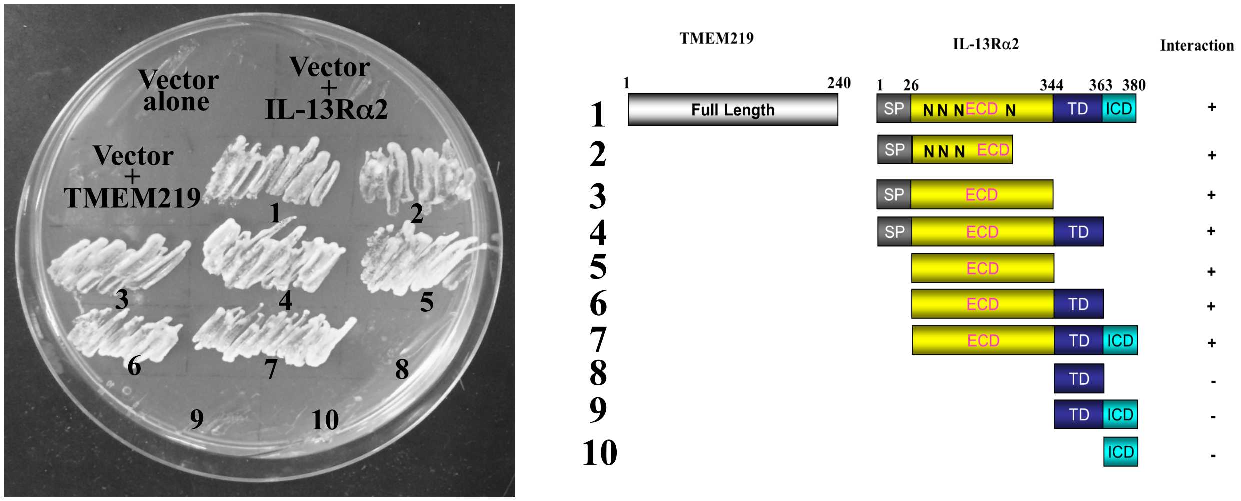

Supplementary Figure 1. BiFC assay for visualization of interaction between TMEM219 and IL-13Rα2. (a) Schematic illustration of TMEM219 and IL-13Rα2 constructs used for BiFC assay. (b, c) FACS evaluation of the 1HAEo (b) and normal human bronchial epithelial (NHBE) (c) cells after two moieties encoding the tagging proteins with each of two Venus YFP fragments (V1 and V2) were used (left panel). The levels of mean fluorescence intensity (MFI) were indicated in the right panel. (d) Yeast two-hybrid complementation assay to determine the major region of IL-13Rα2 interacting with TMEM219. The values in panels b and c represent the mean±SEM of triplicate evaluations in a minimum of three separate experiments. *p<0.05.

Supplementary Figure 2

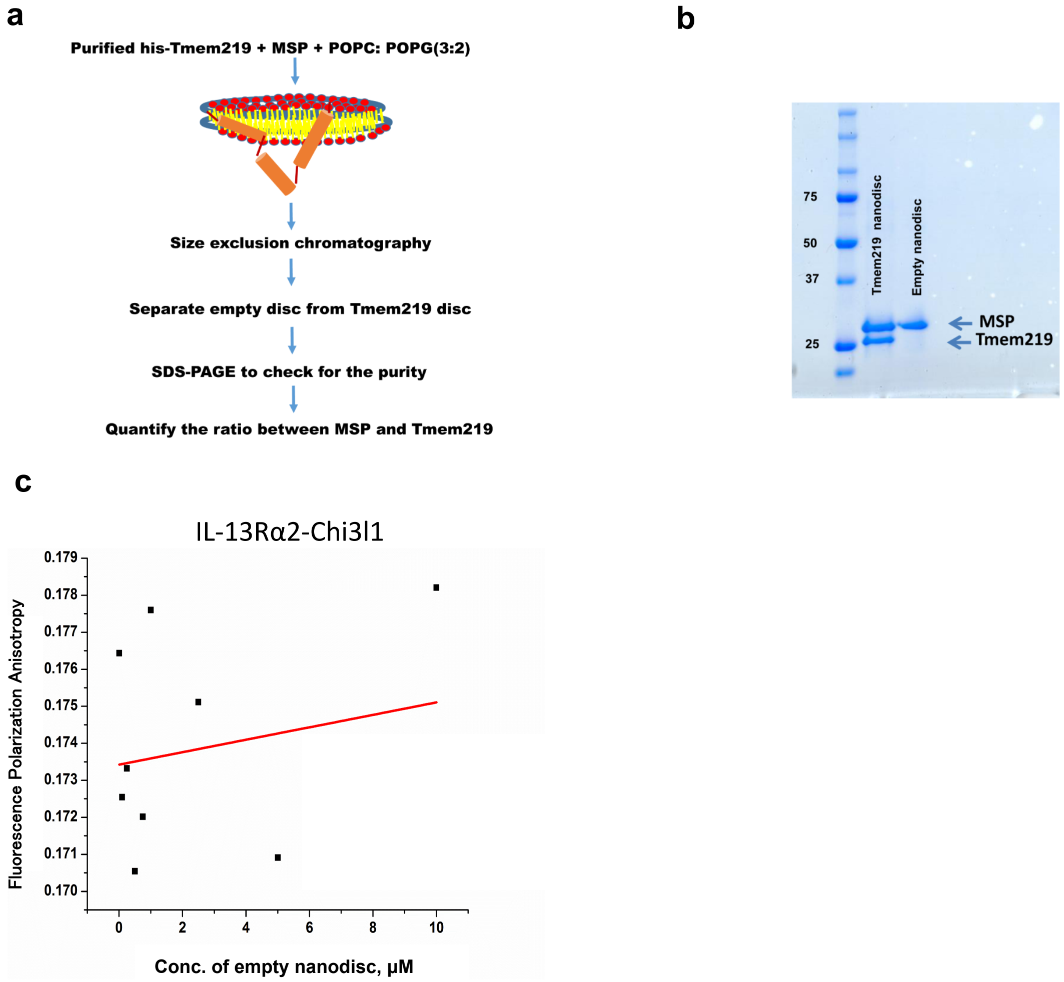

Supplementary Figure 2. TMEM219-nanodisc preparation and control nanodisc and fluorescence anisotropy assays. (a) Flow chart illustrating reconstitution of purified TMEM219-his into ~13 nm nanodisc. (b) TMEM219 was separated from empty nanodisc after size exclusion chromatography using agarose coated nickel beads. Nickel bound proteins represent his tagged TMEM219 nanodisc and flow through represents the empty nanodisc that only contains untagged membrane scaffold protein (MSP). Both of TMEM219 and empty nanodiscs were resolved on SDS-PAGE and stained with coomassie blue for quantification. The stoichiometry between TMEM219 and MSP was determined using ImageJ software. (c). Serial dilution of empty nanodisc (10-10000 nM) were incubated with 10 nM of BODIPY-labeled Chi3l1. No significant increases of fluorescence signals were noted in these empty nanodisc incubations. Samples were run in triplicates and each point represents an average of 60 collected data. Kd values were calculated using Origin 7 software.

Supplementary Figure 3

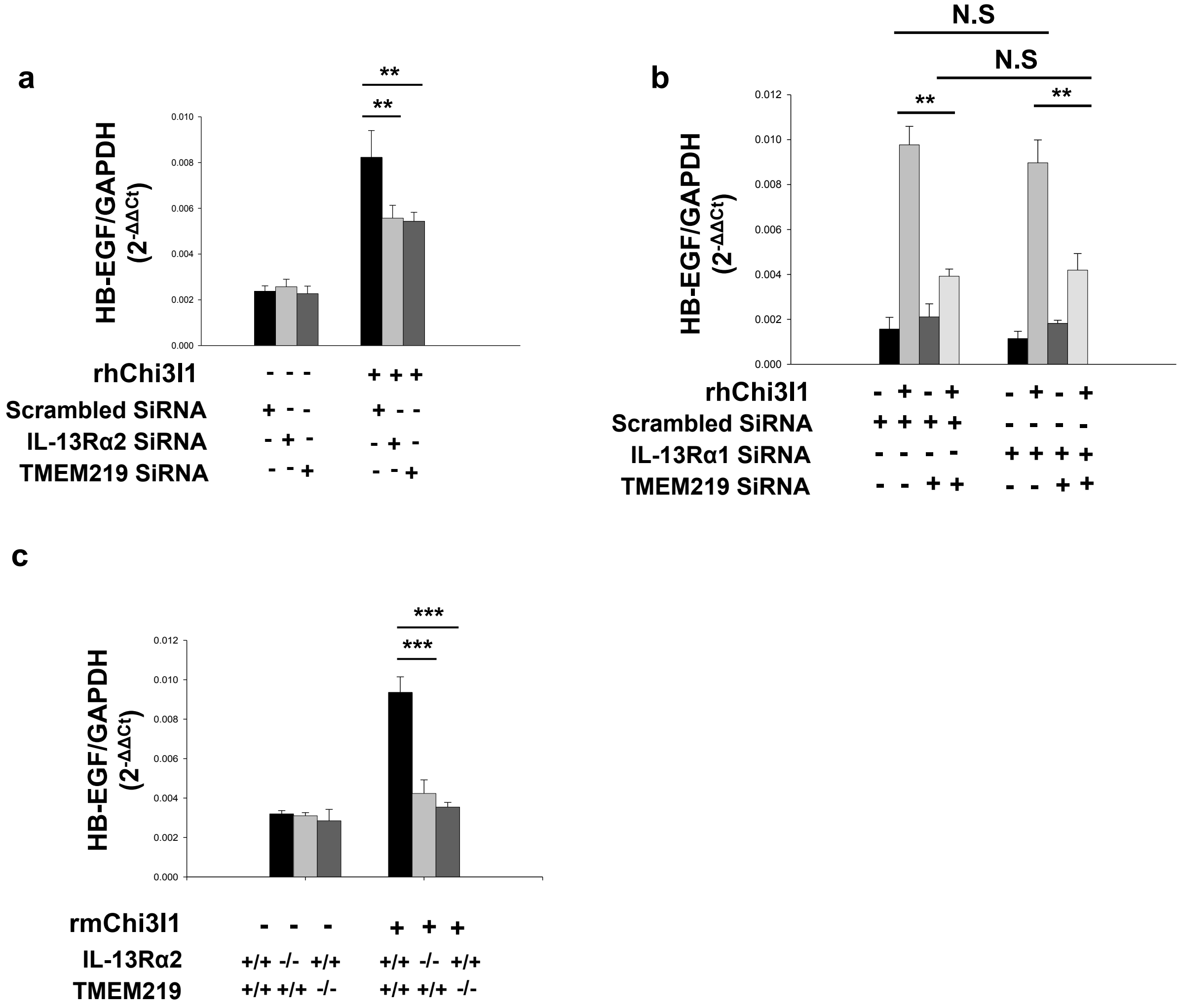

Supplementary Figure 3. TMEM219 and IL-13Rα2 regulation of mRNA expression of Chi3l1-stimulated HB-EGF synthesis in 1HAEo epithelial cells and macrophages. (a and b) The levels of HB-EGF mRNA were evaluated by real time qPCR in 1HAEo cells treated with rChi3l1 (500 ng/ml) with (+) and without (-) siRNA silencing of IL-13Rα2 or TMEM219. (c) The levels of HB-EGF mRNA were evaluated by real time qPCR in peritoneal macrophages from Chi3l1$^{-/-}$ or TMEM219$^{-/-}$ animals after stimulation of the cells with Chi3l1 (500 ng/ml).

Supplementary Figure 4

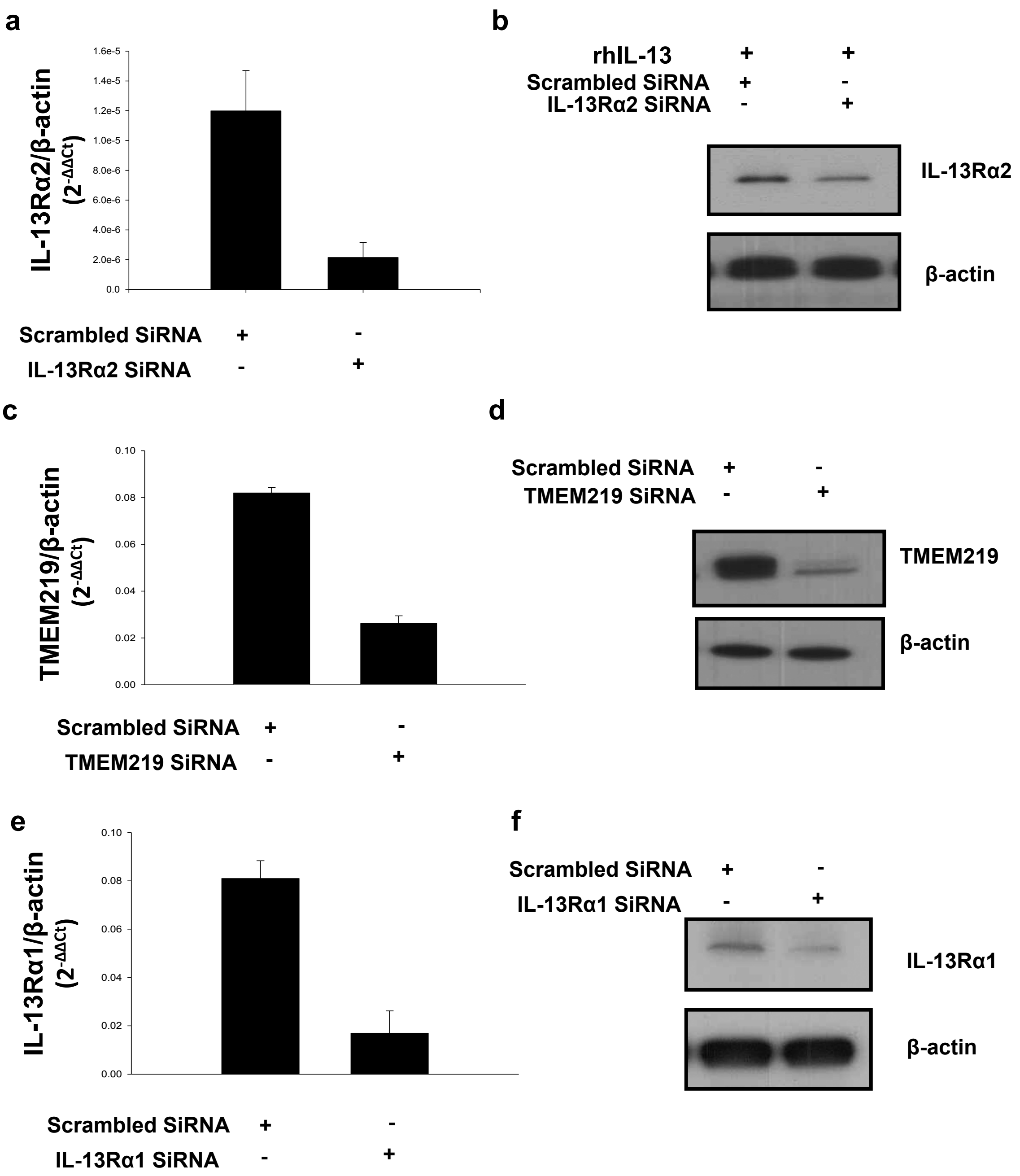

Supplementary Figure 4. siRNA silencing of IL-13Rα2, TMEM219 and IL-13Rα1. (a and b) The levels of 1HAEo IL-13Ra2 mRNA and protein were evaluated by real time qPCR and Western blot evaluations, respectively. The cells were treated with rIL-13 (20 ng/ml) with and without siRNA silencing of IL-13Rα2. (c and d) The levels of 1HAEo TMEM219 mRNA and protein were evaluated by real time qPCR and Western blot evaluations, respectively. (c and d) The levels of 1HAEo TMEM219 mRNA and protein with and without siRNA silencing of TMEM219 were evaluated by real time qPCR and Western blot evaluations, respectively. (e and f) The levels of 1HAEo IL-13Rα1 mRNA and protein with and without siRNA silencing of IL-13Rα1 were evaluated by real time qPCR and Western blot evaluations, respectively.

Supplementary Figure 5

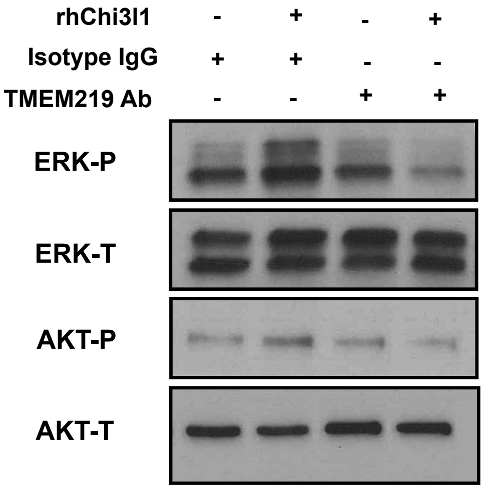

Supplementary Figure 5. TMEM219 plays critical and selective role in rChi3l1-stimulated signaling in lung epithelial cells and macrophages. Western blot evaluations of HB-EGF produced by 1HAEo cells stimulated with rChi3l1 (500 ng/ml) in the presence and absence of TMEM219 neutralizing antibody.

Supplementary Figure 6

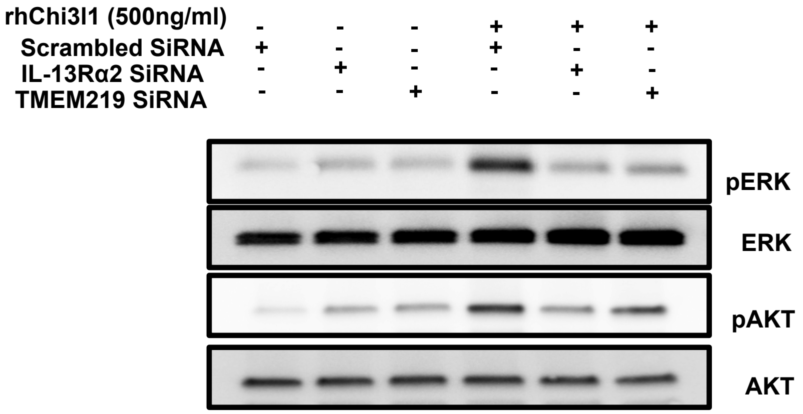

Supplementary Figure 6. Role of IL-13Rα2 and TMEM219 in Chi31l-stimulated signaling and HB-EGF production in normal human bronchial epithelial cells (NHBE). NHBE cells were stimulated with rhChi3L1 (500 ng/ml) with (+) and without (-) the siRNA silencing of IL-13Rα2 or TMEM219 and Western blot evaluations were undertaken to evaluate the activation of the MAPK/ERK and PKB/AKT pathways.

Supplementary Figure 7

a

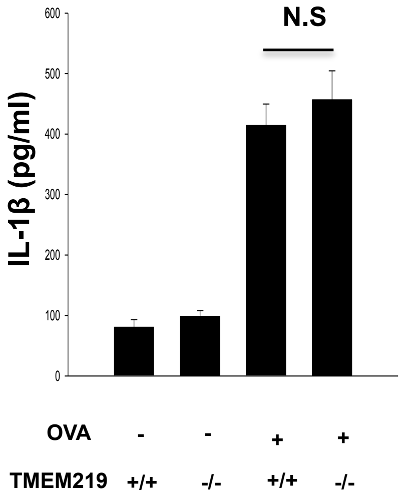

b

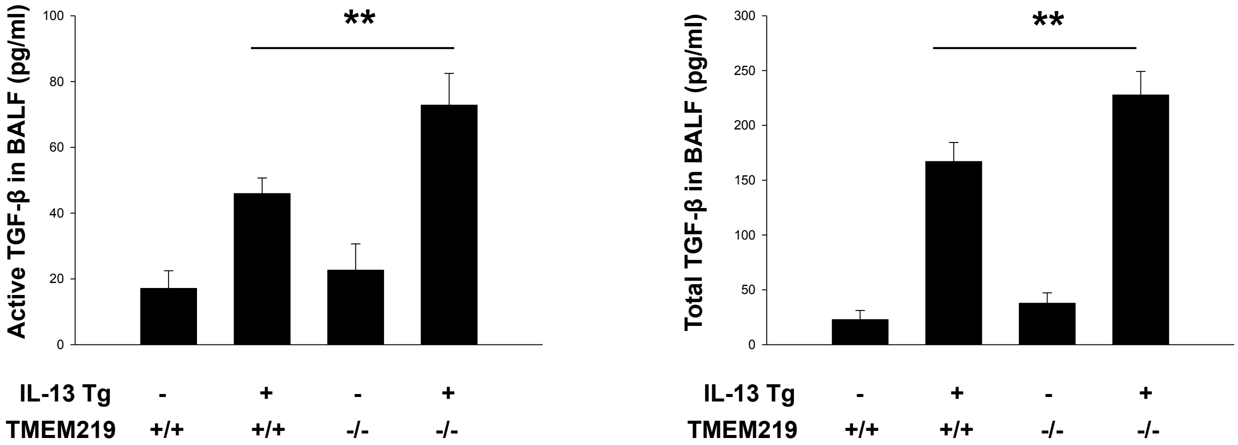

Supplementary Figure 7. TMEM219 regulation of TGF-β expression in the lungs of IL-13Tg mice and IL-1β in the BAL of aeroallergen-challenged mice. (a) The levels of active and total TGF-β in the BAL of WT and IL-13 Tg mice with and without TMEM219 null mutation. (b) The levels of IL-1β detected in the BAL of WT and TMEM219 null mutant mice with and without OVA aeroallergen challenge. The values are the mean±SEM of evaluations on a minimum of 5 mice. **p<0.01, N.S., not significant.

Supplementary Figure 8

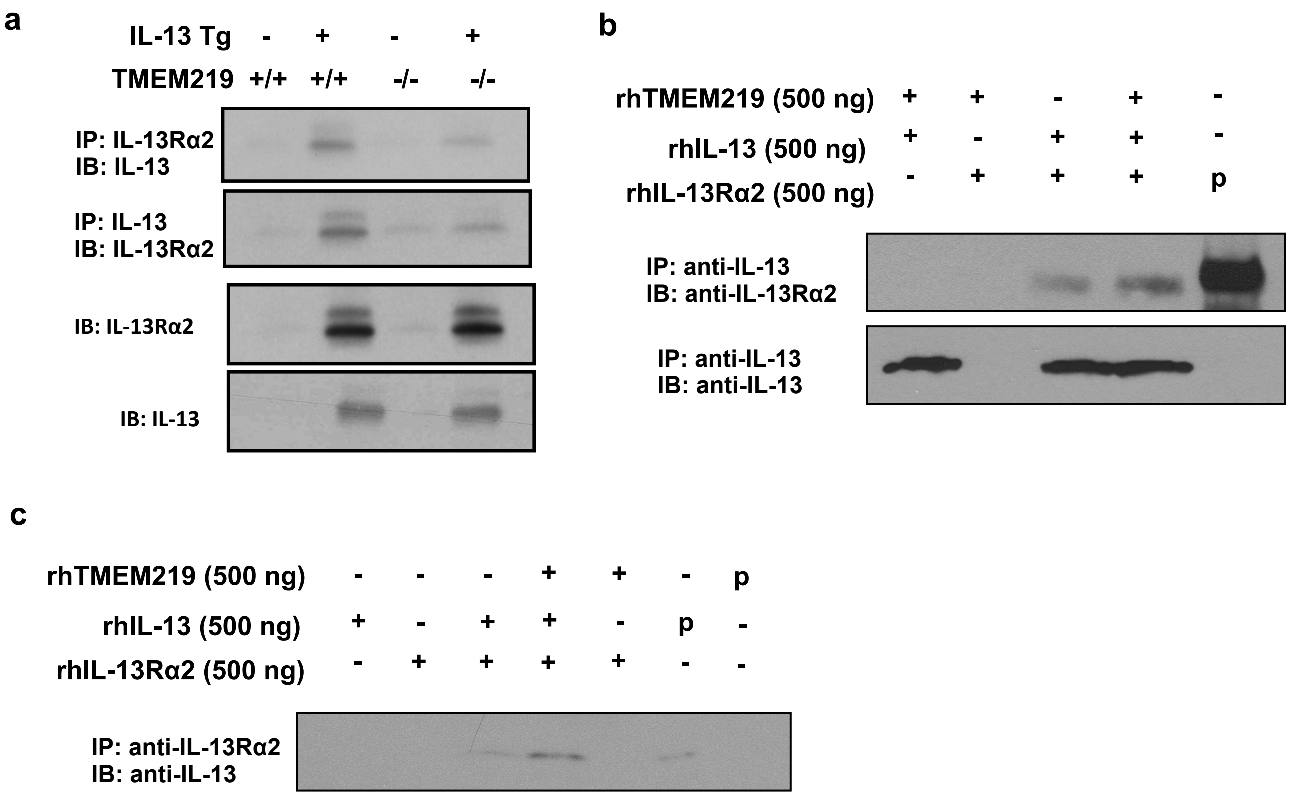

Supplementary Figure 8. Molecular interactions between IL-13, IL-13Rα2 and TMEM219 *in vivo* and *in vitro*. (a) Co-immunoprecipitation (Co-IP) and immunoblot (IB) assay on the lung lysates of WT and IL-13 Tg mice to see interaction of IL-13 and IL-13Rα2 in the presence and absence of TMEM219 null mutation. (b and c) Co-IP and IB assays using recombinant human (rh) IL-13, IL-13Rα2, in the presence and absence of rhTMEM219. P, positive control.

Supplementary Figure 9

A

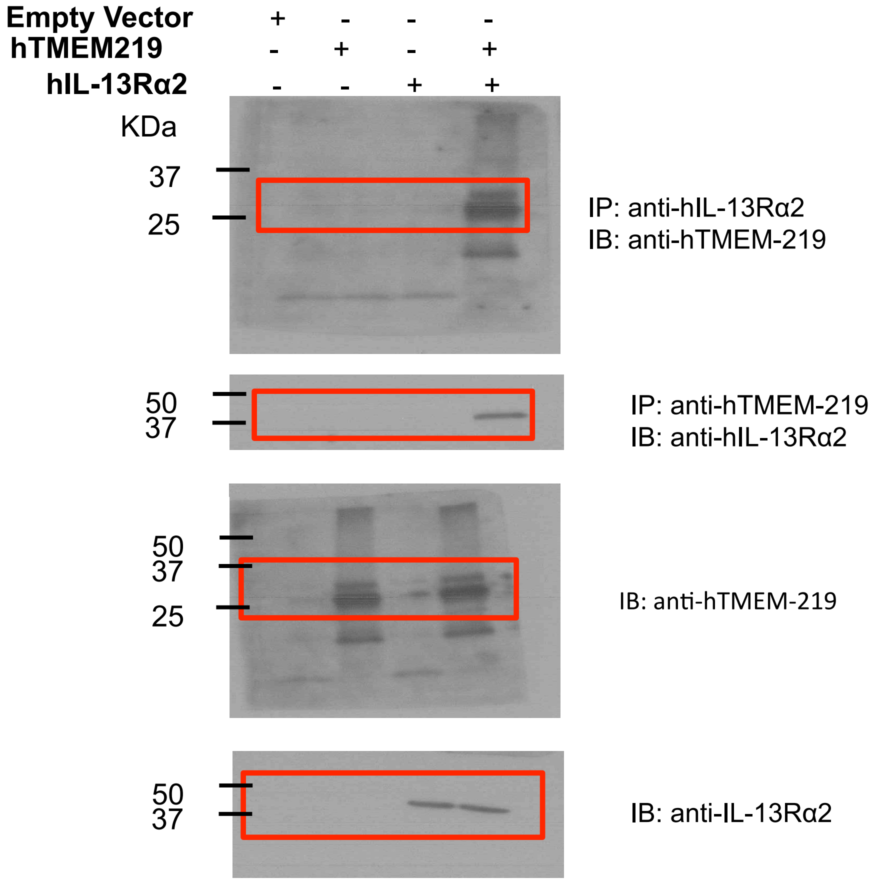

IB: anti-hTMEM-219 – Santacruz (**sc**-244405), 1:500 primary

IB: anti-IL-13Rα2 – R&D (AF146), 1:500 primary

Supplementary Figure 9. -continued

B

**rhTMEM219 (500 ng)**	+	+	-
**rhIL-13 (300 ng)**	-	+	P
**rhIL-13Rα1 (500 ng)**	+	+	P

IP: anti-TMEM219
IB: anti-IL-13Rα1

IP: anti-TMEM219
IB: anti-IL-13

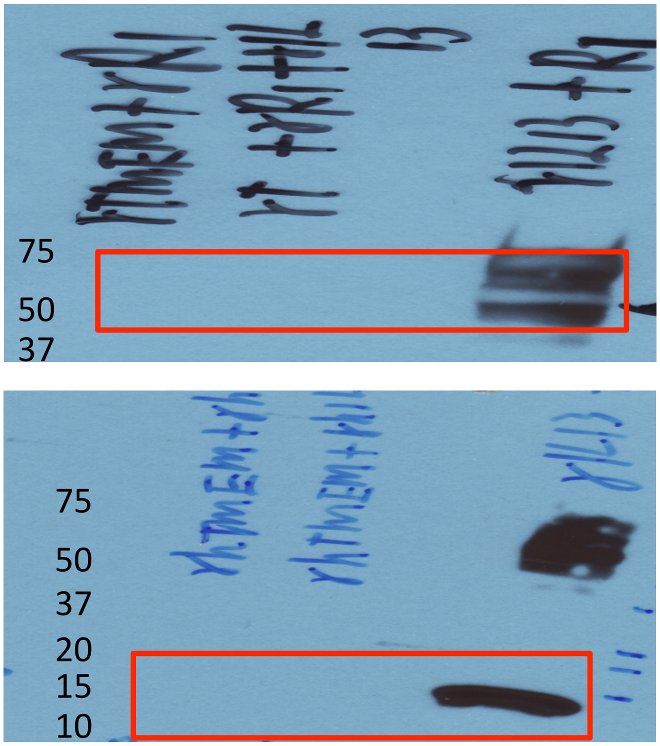

IB: anti-IL-13Ra1 – R&D (**MAB1462**), 1:500 primary
IB: anti-IL-13– sR&D (MAB413), 1:200 primary

Supplementary Figure 9. -continued

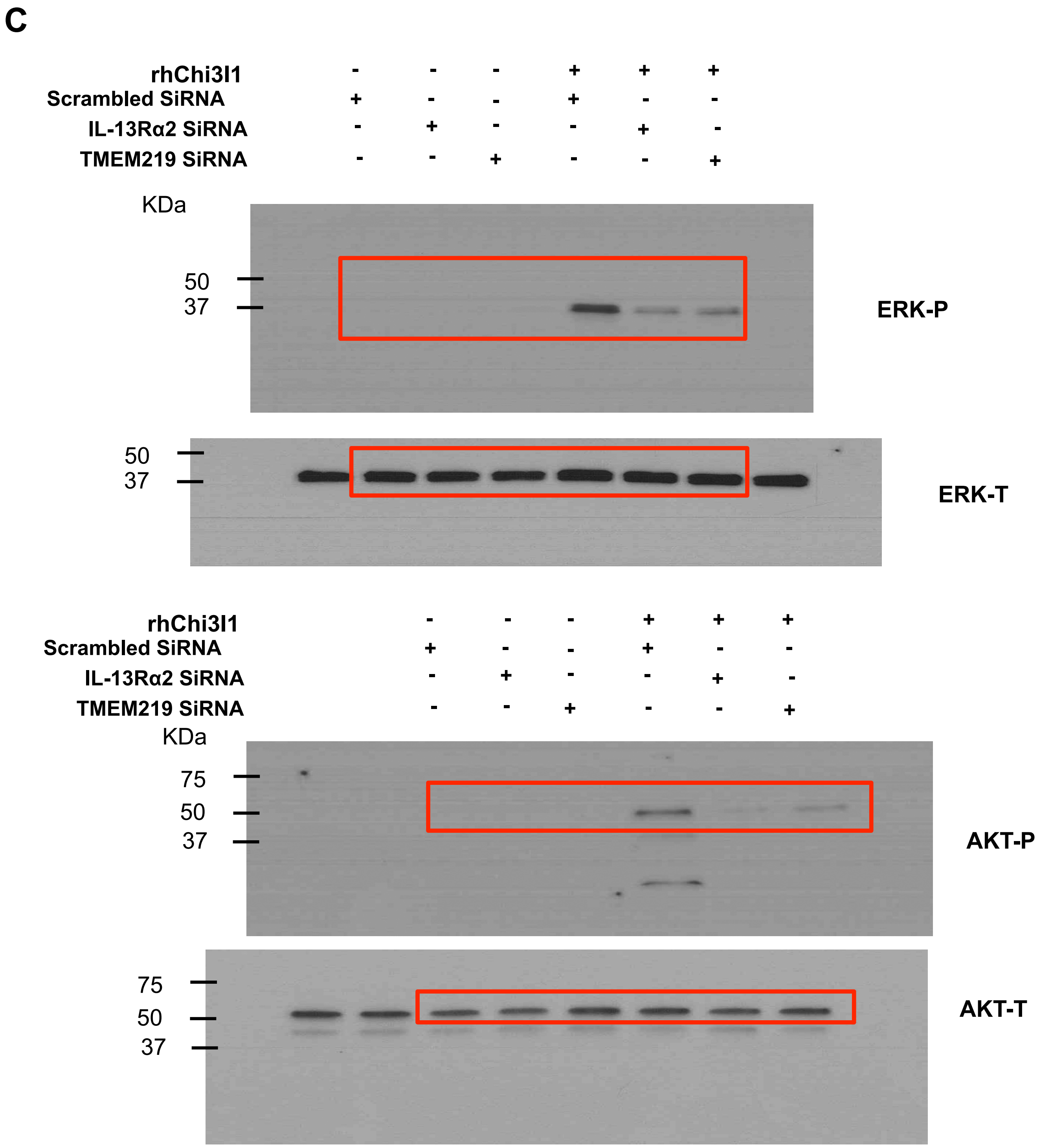

pERK – Cell signaling (**9101S**), 1:1000 primary
ERK - Cell signaling (**9102**), 1:1000 primary
pAKT – Cell signaling (**4058**), 1:1000 primary
AKT – Cell signaling (**9272**), 1:1000 primary

Supplementary Figure 9. -continued

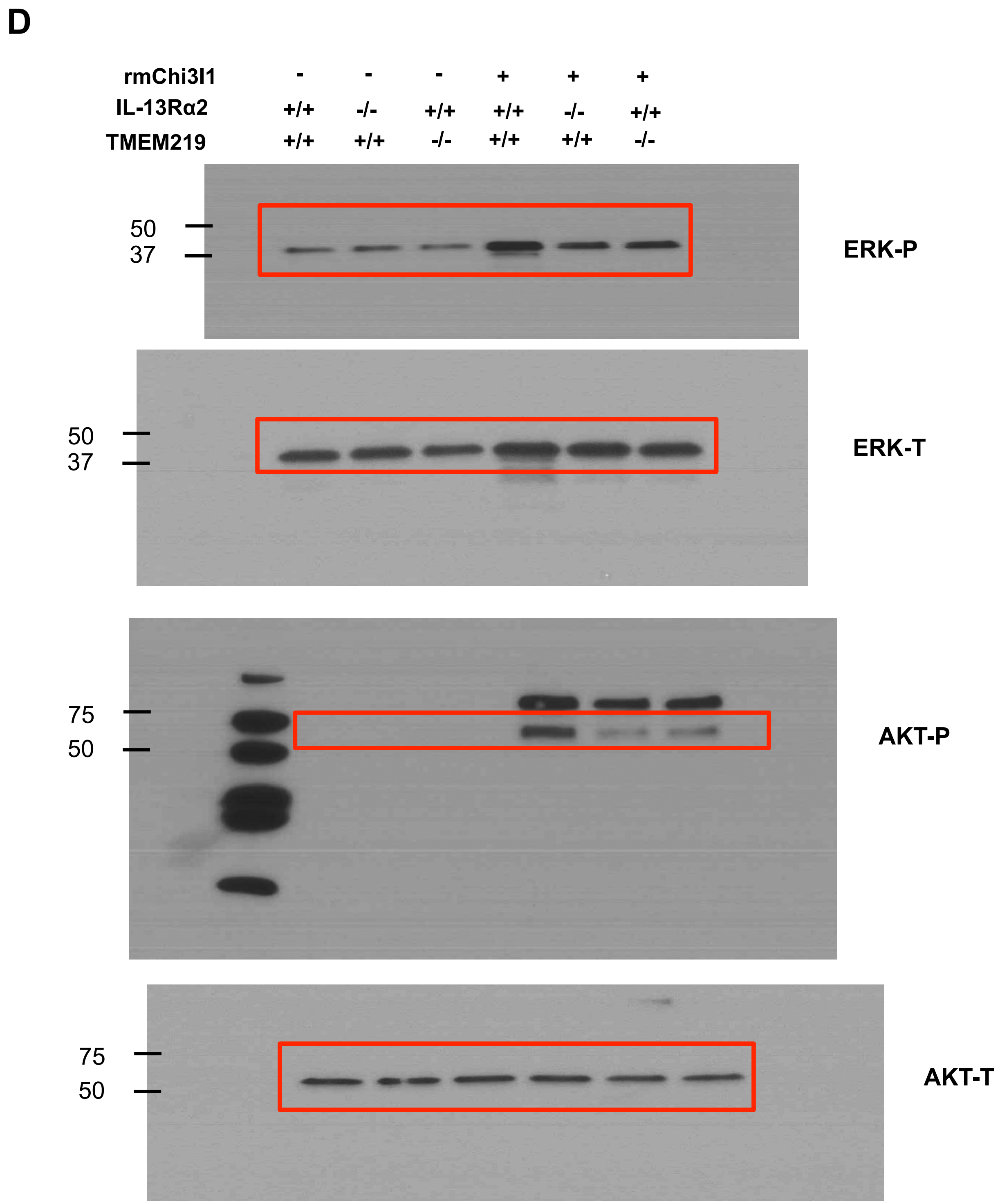

pERK – Cell signaling (**9101S**), 1:1000 primary
ERK - Cell signaling (**9102**), 1:1000 primary
pAKT – Cell signaling (**4058**), 1:1000 primary
AKT – Cell signaling (**9272**), 1:1000 primary

Supplementary Figure 9. -continued

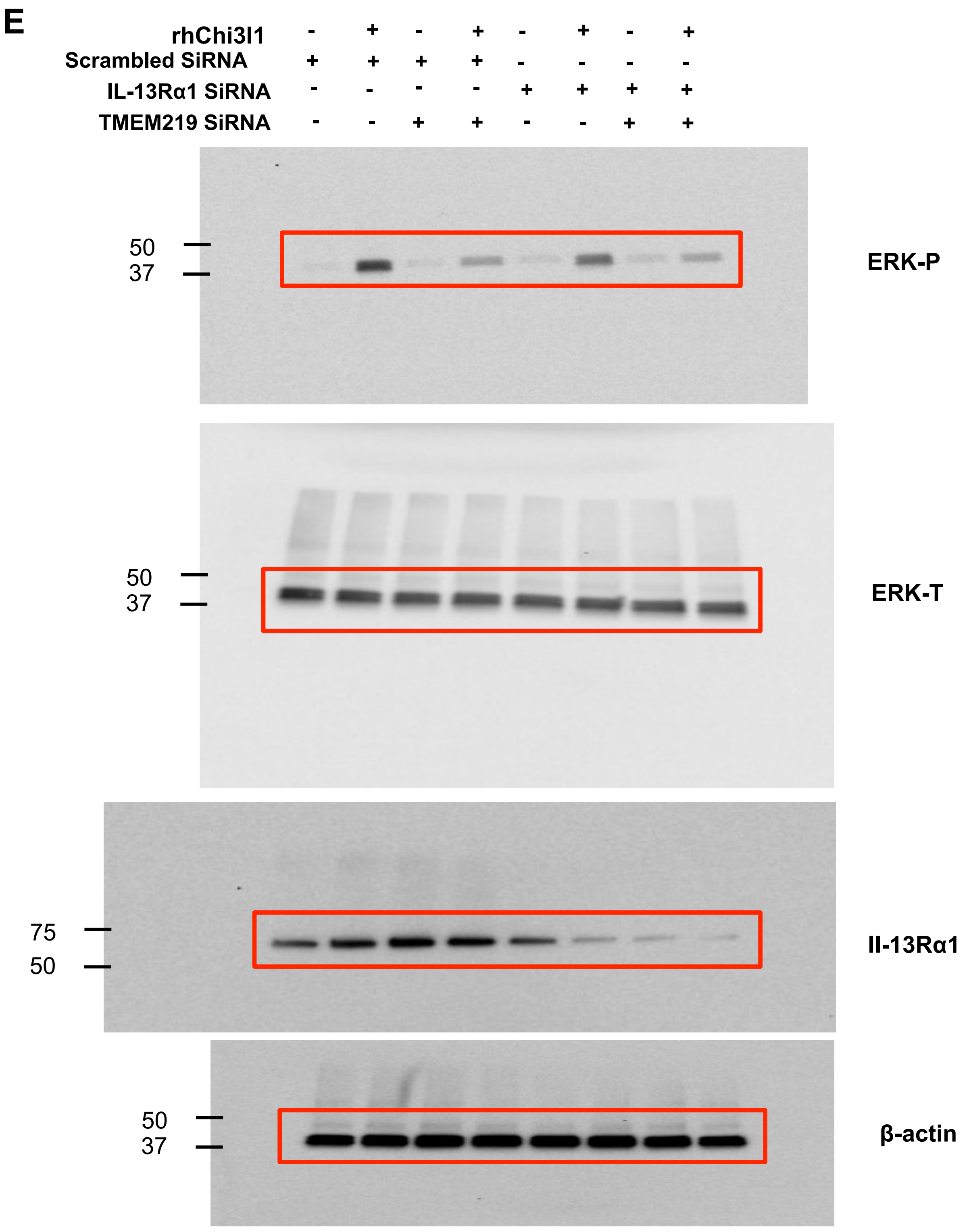

pERK – Cell signaling (**9101S**), 1:1000 primary
ERK - Cell signaling (**9102**), 1:1000 primary
Il-13Ra1 – R&D (**MAB1462**), 1:500 primary
β-actin– santacruz (**SC-47778**), 1:3000 primary

Supplementary Figure 9. -continued

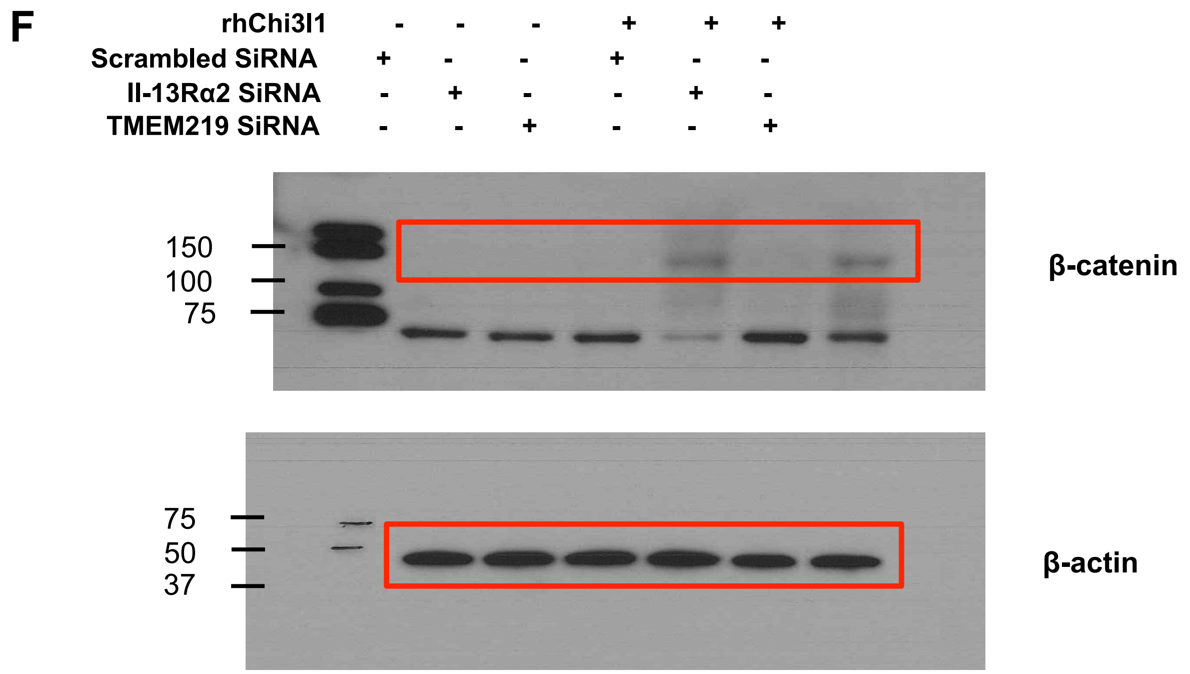

β-catenin – santacruz (**SC-7963**), 1:200 primary
β-actin– santacruz (**SC-47778**), 1:3000 primary

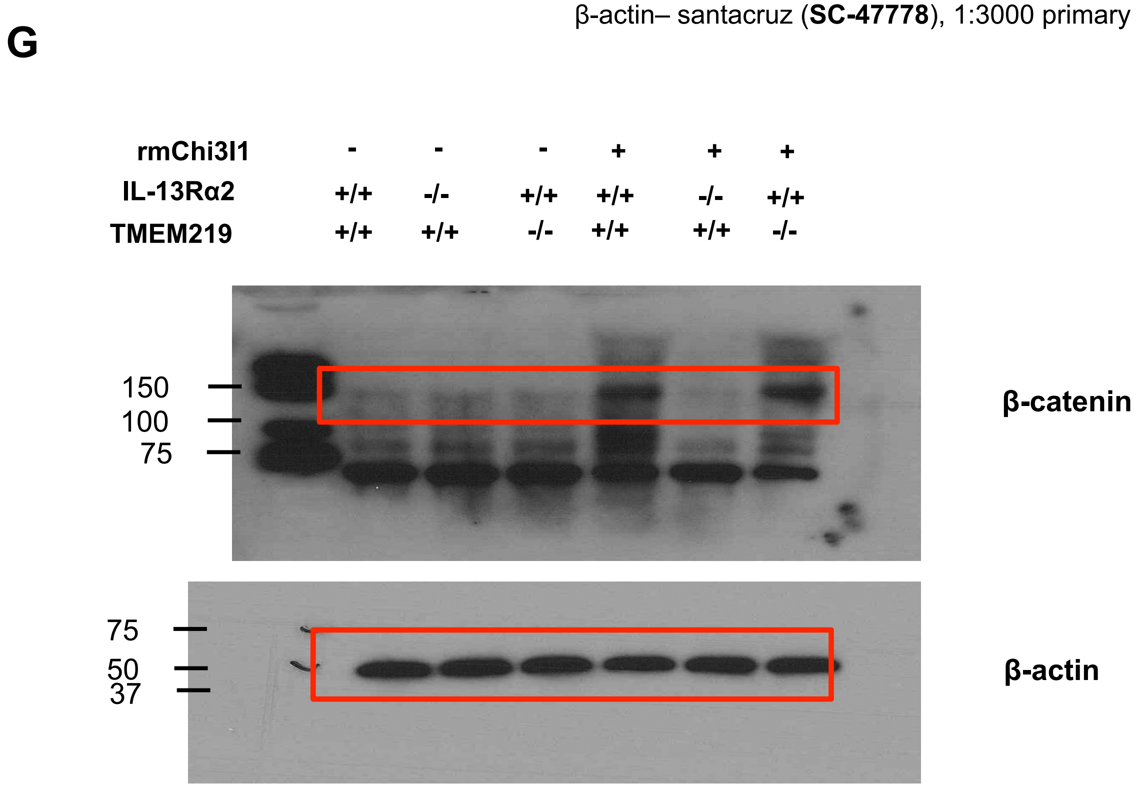

β-catenin – santacruz (**SC-7963**), 1:200 primary
β-actin– santacruz (**SC-47778**), 1:3000 primary

Supplementary Figure 9. -continued

H

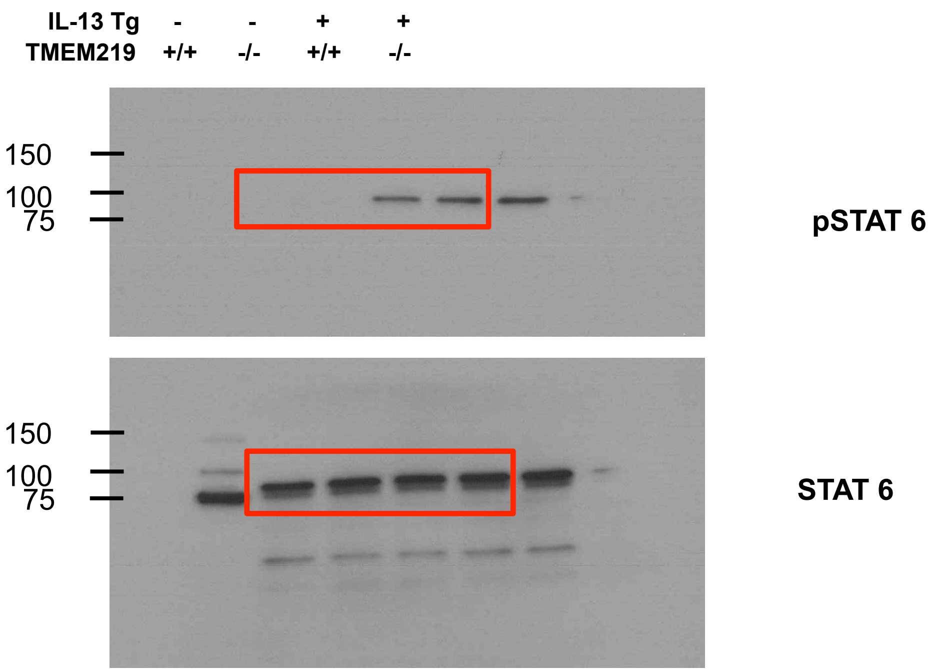

P-stat 6– Emdmillipore (**05-590**), 1:500 primary
Stat 6– cell signaling (**9362S**), 1:500 primary

Supplementary Figure 9. -continued

I

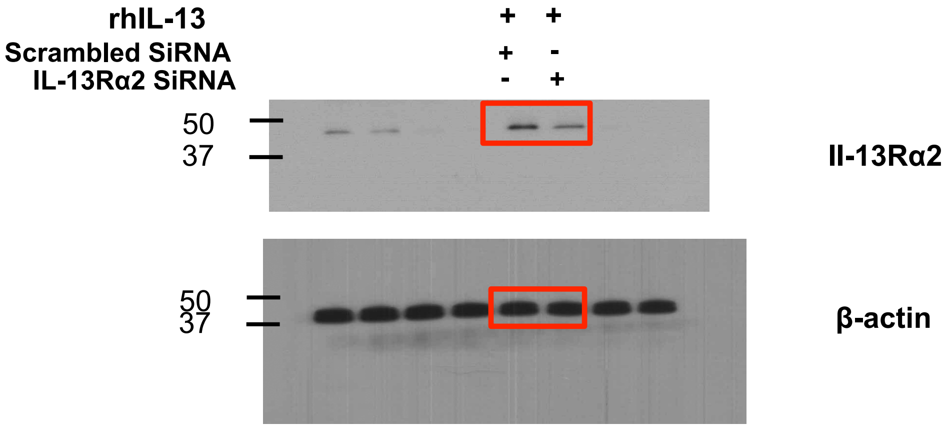

IL-13Rα2 – R&D (AF146), 1:500 primary
β-actin– santacruz (**SC-47778**), 1:3000 primary

J

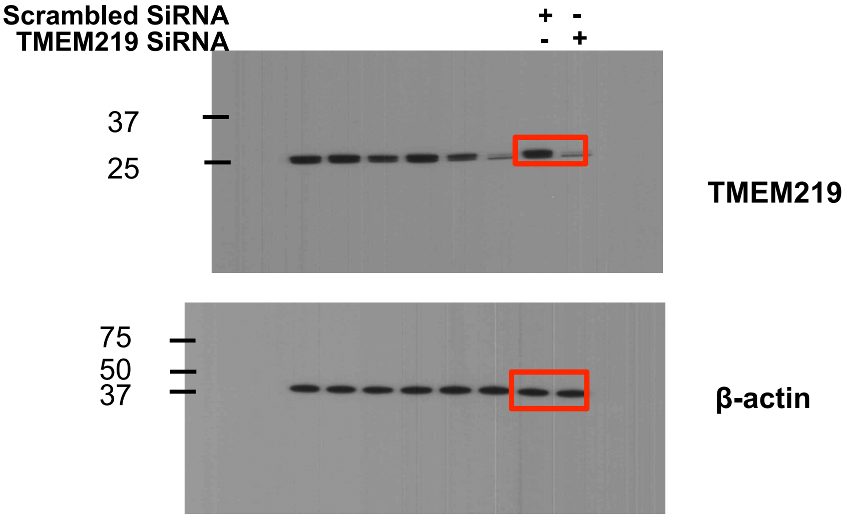

TMEM219 – Santacruz (**sc-**244405), 1:500 primary
β-actin– santacruz (**SC-47778**), 1:3000 primary

K

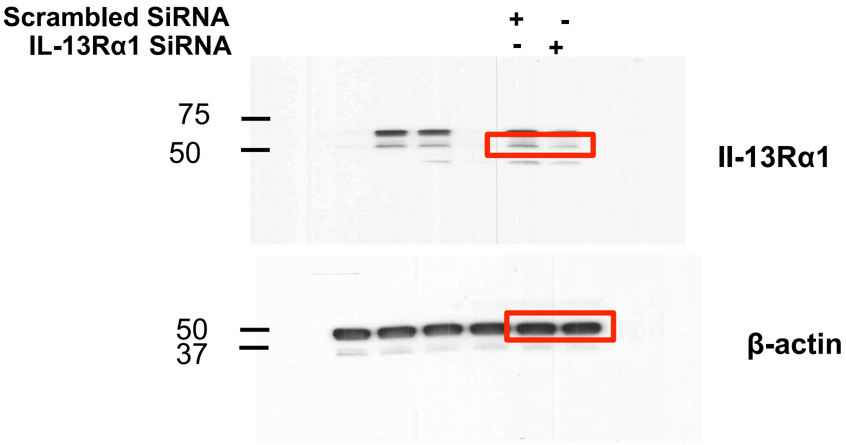

Il-13Ra1 – R&D (**MAB1462**), 1:500 primary
β-actin– santacruz (**SC-47778**), 1:3000 primary

L

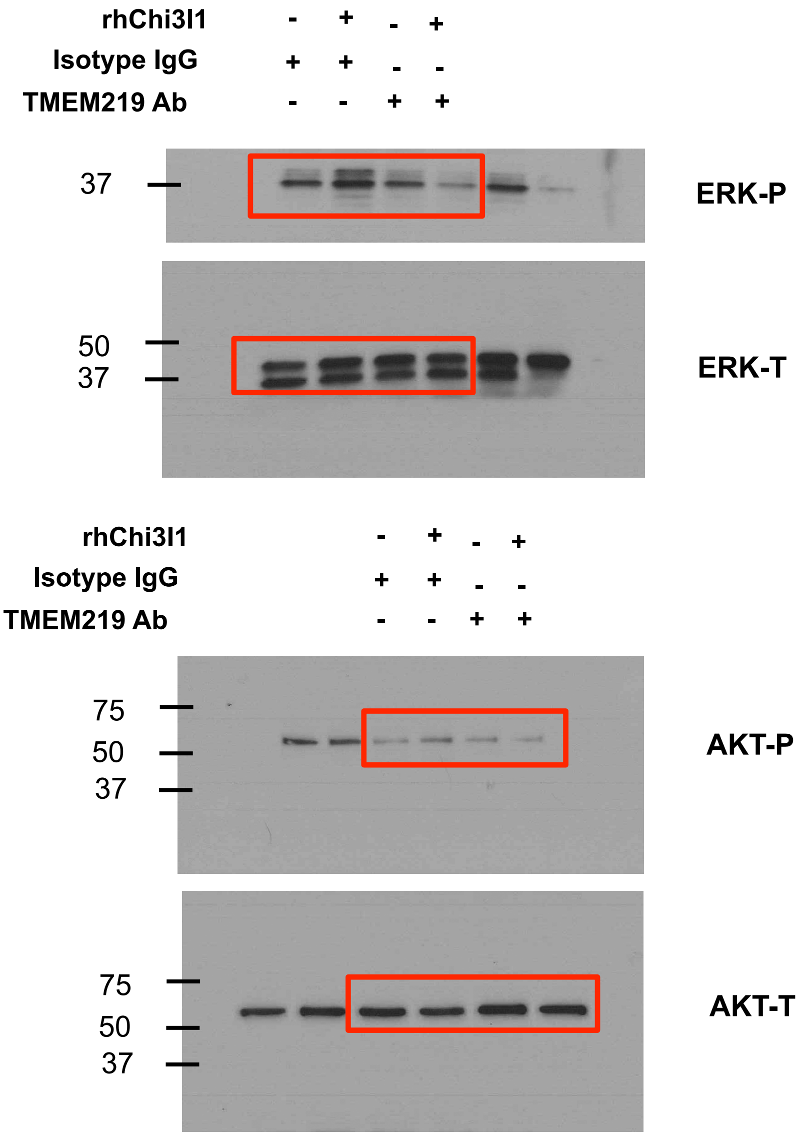

pERK – Cell signaling (**9101S**), 1:1000 primary
ERK - Cell signaling (**9102**), 1:1000 primary
pAKT – Cell signaling (**4058**), 1:1000 primary
AKT – Cell signaling (**9272**), 1:1000 primary

Supplementary Figure 9. -continued

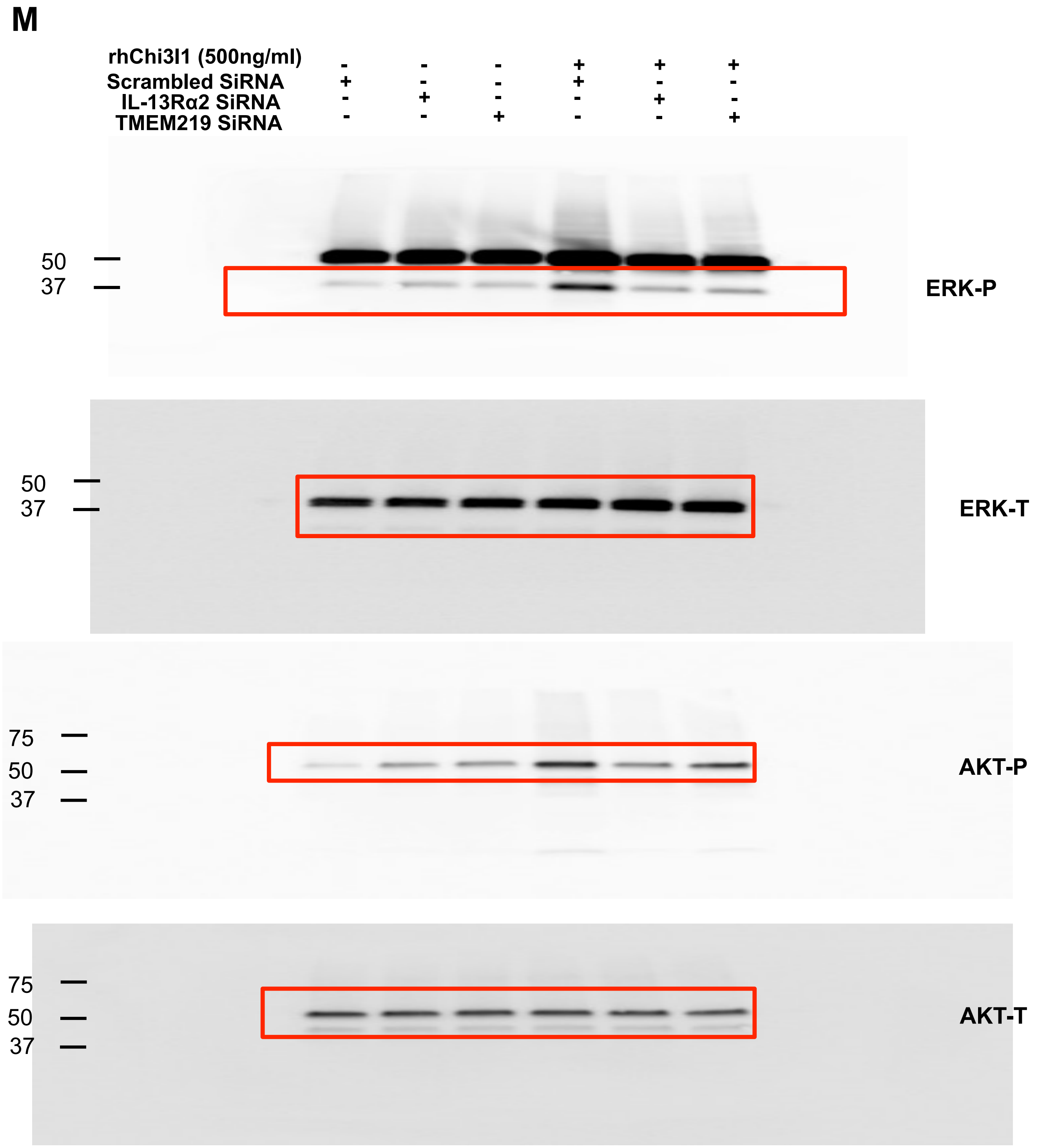

pERK – Cell signaling (**9101S**), 1:1000 primary
ERK - Cell signaling (**9102**), 1:1000 primary

pAKT – Cell signaling (**4058**), 1:1000 primary
AKT – Cell signaling (**9272**), 1:1000 primary

Supplementary Figure 9. -continued

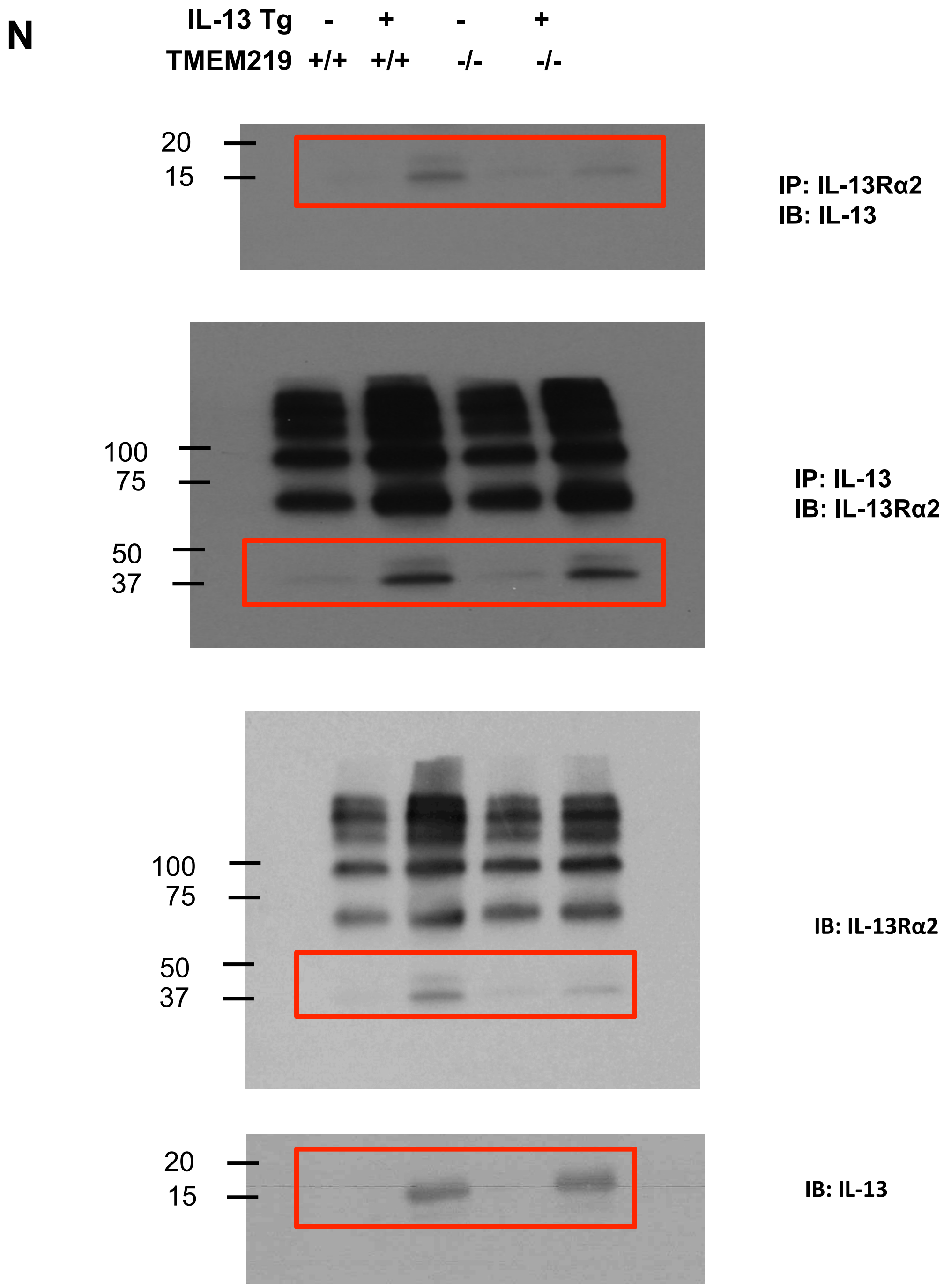

IL-13Rα2 – R&D (AF146), 1:500 primary
IL-13– sR&D (MAB413), 1:200 primary

Supplementary Figure 9. -continued

O

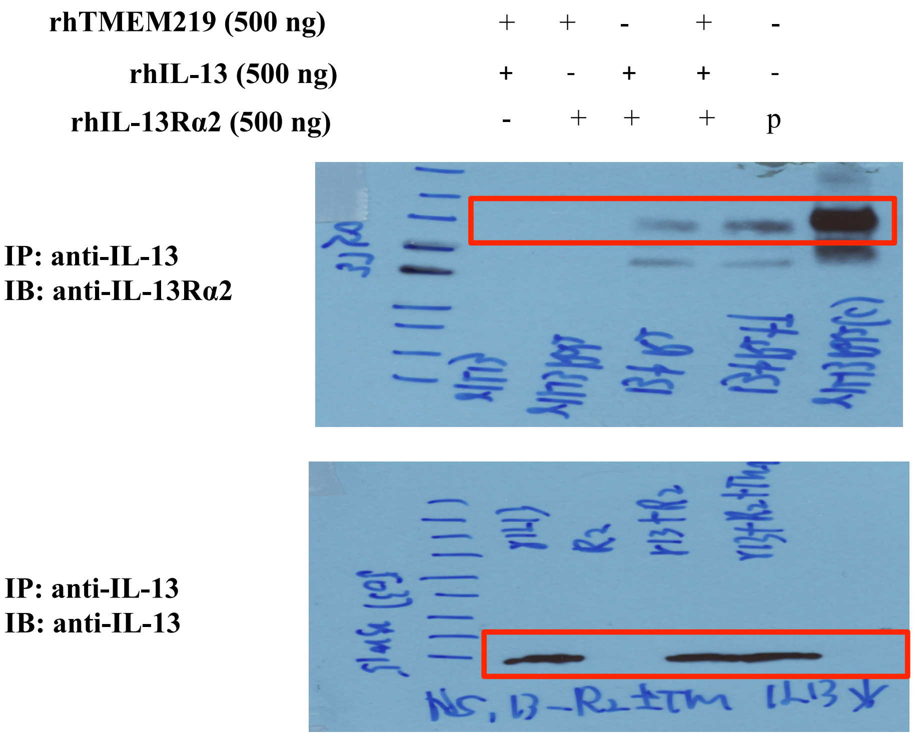

IL-13Rα2 – R&D (AF146), 1:500 primary
IL-13– sR&D (MAB413), 1:200 primary

P

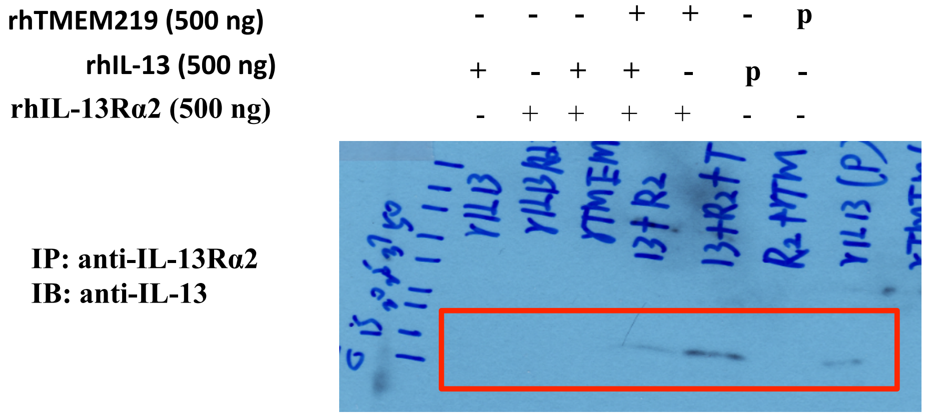

IL-13– sR&D (MAB413), 1:200 primary

Supplementary Figure 9. Full immunoblots and autoradiography films. (**A-M**) Original images of the blots shown in Figs. 1b (**A**), 1c (**B**), 4a (**C**), 4b (**D**), 4c (**E**), 4d (**F**), 4e (**G**), 6k (**H**), and in Supplementary Figs. 4b (**I**), 4d (**J**), 4f (**K**), 5 (**L**) 6 (**M**), 8a (**N**), 8b (**O**) and 8c (**P**). Cropped areas are marked by Red lines.
